# Enhancing the Red and Near Infrared OLED Efficiency of a TADF Emitter through an Internal Solvation Effect

**DOI:** 10.1021/acsami.6c01458

**Published:** 2026-04-29

**Authors:** Wojciech Derkowski, Piotr Pander, Adam Zuba, Krzysztof Durka, Sergiusz Luliński, Andrew P. Monkman

**Affiliations:** a Faculty of Chemistry, Warsaw University of Technology, Noakowskiego 3, Warsaw 00-664, Poland; b Faculty of Chemistry, Silesian University of Technology, Strzody 9, Gliwice 44-100, Poland; c Centre for Organic and Nanohybrid Electronics, Silesian University of Technology, Konarskiego 22B, Gliwice 44-100, Poland; d Department of Physics, 3057 Durham University, South Road, Durham DH1 3LE, U.K.

**Keywords:** TADF, OLED, solid state solvation, near-infrared luminescence, triarylboron compound

## Abstract

We report thermally activated delayed fluorescence (TADF) emitters, namely, **PTZ-Dipp-SO2B**, and **PTZ-Dipp-(Bu)­SO2B**, featuring an additional *n*-butyl group on the 10*H*-dibenzo­[*b*,*e*]­[1,4]­thiaborinine 5,5-dioxide (SO2B) core enhancing its solid-state luminescence through an internal solvation effect. In dilute low-polarity solutions, both molecules show a yellow-orange photoluminescence with λ_em_ = 561–588 nm and red to near-infrared (NIR) PL in neat films, λ_em_ = 680–706 nm. The PL of **PTZ-Dipp-(Bu)­SO2B** is systematically blue-shifted with respect to **PTZ-Dipp-SO2B** thanks to a ∼0.1 eV difference in LUMO energy caused by the σ-donor properties of the *n*-butyl group. These molecules were successfully applied as luminescent dopants to organic light-emitting diodes (OLEDs). Although both compounds have similar electronic structures and exhibit comparable Φ_PL_ in solution, the **PTZ-Dipp-(Bu)­SO2B** is a significantly more efficient luminophore in the solid state than its counterpart. The OLED external quantum efficiency (EQE) at 7% load is 12.2% and in the neat film is 1.0% for **PTZ-Dipp-(Bu)­SO2B**, while those for **PTZ-Dipp-SO2B** are 5.4% and 0.4%, respectively. The difference in λ_em_ or λ_EL_ between the two emitters is too small to explain the differences in EQE exclusively with the effects of the energy gap law. Instead, we believe that the *n*-butyl group disrupts the environment around the molecule through locally lowering the polarity and disturbing the formation of aggregates in the solid state. From previous observations of solvation of the emitter with hexane molecules, we term this phenomenon as the internal solvation effect.

## Graphical abstract

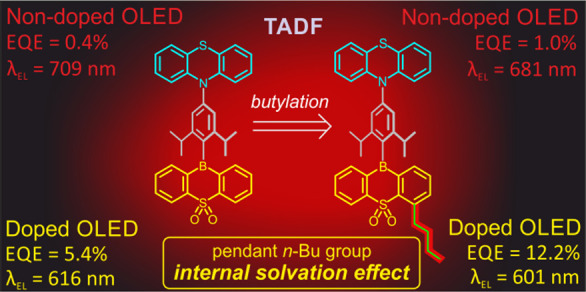

## Introduction

Organic light-emitting diodes (OLEDs) have been enthusiastically investigated in recent years and are currently ubiquitous in high-end luminescent displays of many consumer electronic devices.^1–3^ OLEDs are flexible and display vastly improved color performance with respect to conventional liquid crystal displays (LCD), also providing high electroluminescent outputs while being energy-efficient.^1,3–5^ From the standpoint of device efficiency, emitters able to effectively harvest triplet excitons such as phosphorescent Pt­(II) and Ir­(III) complexes^6–9^ and thermally activated delayed fluorescence (TADF) molecules are of high importance.^10–14^ Near infrared (NIR) emitters and OLEDs are particularly valuable due to their crucial applications spanning from optical communications to biomedical sensors, photodynamic therapy (PDT), night vision, and more generally security applications to name a few.^1,15–17^ Currently, the best NIR OLEDs are based on Pt­(II) complexes, but there is a strong demand for precious metal-free alternatives, of which the best are TADF emitters. Typically, NIR TADF emitters feature D–A architecture composed of strong electron donating and withdrawing groups, which helps to decrease the HOMO–LUMO energy gap and minimize the excited singlet–triplet energy splitting (Δ*E*_ST_).^18–22^ To simplify the construction of OLEDs and further shift the luminescence toward the NIR, TADF emitters are often employed as neat emissive layers in nondoped OLED devices.^23,24^ Since the aggregation effects in the emitting layers can significantly alter the photophysical properties of the particular system, controlling the intermolecular interactions constitutes an important matter worthy of detailed study. Recent examples demonstrate that device performance can be improved by disrupting aggregation through functionalization of the emitter molecule with bulky substituents.^25–29^

Generally speaking, solid state solvation effects (SSSE) are analogous to the interactions between solvent and solute in solutions, but occurring in solids.^30^ In the case of this work, the emitter molecule (solute) is surrounded by other molecules (solvent), be it host molecules (doped film) or other emitter molecules (neat film). These solvating polar molecules can therefore stabilize the CT states of D–A type TADF emitters. In our work, we demonstrate how SSSE can be perturbed and suppressed, minimizing their detrimental effects on NIR CT luminescence.

Herein, we present the D−π–A organoboron emitter **PTZ-Dipp-SO2B** and its analogue **PTZ-Dipp-(Bu)­SO2B** bearing an *n*-butyl group attached to a 10*H*-dibenzo­[*b*,*e*]­[1,4]­thiaborinine 5,5-dioxide (SO2B) core. We demonstrate that incorporation of the *n*-butyl group results in a 2–3-fold increase of device efficiency and likewise in photoluminescence efficiency in the solid state. Considering the crystallographic analysis, we ascribe this strong effect to the weakening of intermolecular interactions. Skabara et al. have recently observed a somewhat similar effect in the crystalline state of a TADF emitter solvated with hexane.^25^ This solvation effect of hexane can be interpreted as analogous to that of the attached *n*-butyl group, and hence, we propose to introduce the term internal solvation effect (ISE) to describe the behavior of the studied **PTZ-Dipp-(Bu)­SO2B** emitter. Thus, it appears that this internal solvation effect offers an important and effective tool for improving the NIR OLED efficiency.

## Results and Discussion

### Synthesis

The synthesis of **PTZ-Dipp-SO2B** and **PTZ-Dipp-(Bu)­SO2B** essentially followed the procedure reported recently for related emitters bearing carbazole or phenoxazine donors.^31^ However, 10-chloro-10*H*-dibenzo­[*b*,*e*]­[1,4]­thiaborinine 5,5-dioxide **1** was not converted into the 10-methoxy derivative but to the 10-isopropoxy ester **2**, which is more conveniently processed due to its higher solubility in THF (Figure 1). A solution of **2** in THF was then added to a solution of the aryllithium derivative **4** generated from the respective aryl bromide **3** by Br–Li exchange with *n*-BuLi. It appears that the boron arylation is relatively slow, which may be rationalized by steric hindrance enhanced by the presence of a rather bulky O-*i*-Pr group. In these conditions, the SO2B group undergoes *ortho*-directed deprotonation at the 4-position with **4** due to a strong acidifying effect of the sulfonyl group. This is followed by alkylation of the generated aryllithium intermediate with *n*-BuBr, a byproduct of Br–Li exchange, eventually leading to **PTZ-Dipp-(Bu)­SO2B**. Compounds **PTZ-Dipp-SO2B** and **PTZ-Dipp-(Bu)­SO2B** could easily be separated by column chromatography and finally isolated in reasonable yields (28 and 24%, respectively). It should be noted that an attempted synthesis of **PTZ-Dipp-(Bu)­SO2B** by deprotonative lithiation of **PTZ-Dipp-SO2B** with LDA or *t*-BuLi followed by trapping with *n*-BuI gave a very low yield of the butylated analogue. The formation of the desired products was confirmed by multinuclear NMR, HRMS, and X-ray crystallography. Both products are stable under ambient conditions and exhibit high thermal stability desired for OLED fabrication. TGA analysis reveals their decomposition temperatures *T*_d_ (corresponding to 5% weight loss) at 280 and 295 °C for **PTZ-Dipp-SO2B** and **PTZ-Dipp-(Bu)­SO2B**, respectively (Figures S19 and S20 in the Supporting Information). Notably, despite its higher molecular weight, **PTZ-Dipp-(Bu)­SO2B** melts at a much lower temperature (*T*_m_ = 213 °C) with respect to **PTZ-Dipp-SO2B** (*T*_m_ = 240 °C).

**Figure 1:**
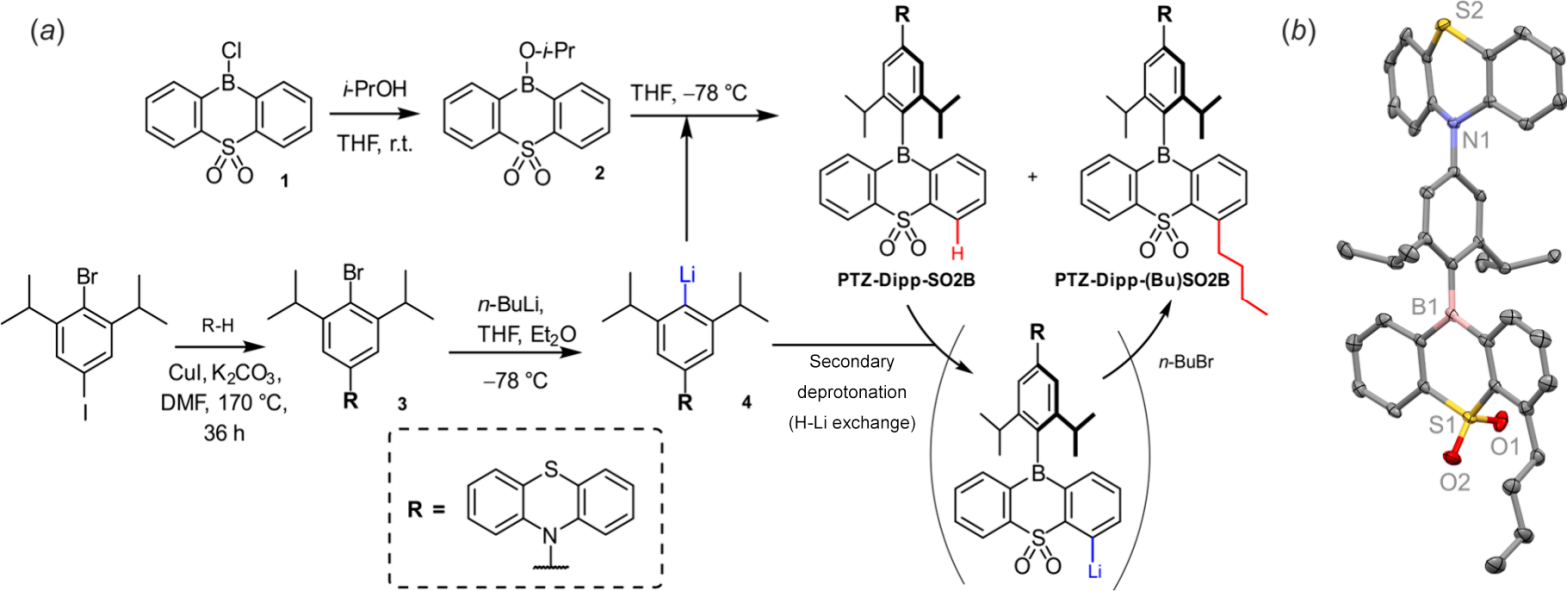
(a) Synthesis of **PTZ-Dipp-SO2B** and **PTZ-Dipp-(Bu)­SO2B**. (b) Molecular structure of **PTZ-Dipp-(Bu)­SO2B** determined by single-crystal X-ray diffraction (ellipsoids drawn at the 50% probability level; H atoms are omitted for clarity).

### X-ray Structures

**PTZ-Dipp-SO2B** and **PTZ-Dipp-(Bu)­SO2B** crystallize in monoclinic *P*2_1_ and orthorhombic *P*2_1_2_1_2_1_ space groups, respectively, with one molecule in the asymmetric part of the unit cell; the former structure additionally contains one DCM solvent molecule. In both compounds, the phenothiazine and SO2B moieties are almost perpendicular to the Dipp spacer. They display restricted conformation flexibility reflected by some deviations of the dibenzothiaborinine dioxide moiety from planarity (Figure S1, SI).^32,33^ In **PTZ-Dipp-(Bu)­SO2B**, the phenothiazine fragment adopts a pronounced boat conformation, whereas in the case of **PTZ-Dipp-SO2B**, PTZ remains nearly planar. Crystal packing in all studied structures is governed by weak C–H···C­(π), C–H···O, and C–H···S intermolecular interactions. Molecules of **PTZ-Dipp-SO2B** form a 2D highly ordered double-herringbone crystal pattern, with PTZ and SO2B moieties organized into parallel chains propagated along the [010] direction (Figure 2). The interactions between SO2B moieties are further supported by hydrogen C–H···O and halogen-type O···Cl bonds with DCM molecules. Despite higher crystal symmetry, the basic crystal motif in **PTZ-Dipp-(Bu)­SO2B** is reduced to a 1D molecular chain with C–H···C­(π) and C–H···S interactions formed between PTZ units, supported by C–H···O hydrogen bonds between *i*-Pr and SO2B groups. In addition, π-stacking interactions occur between aromatic rings belonging to PTZ and SO2B units; however, the resulting face-to-tail dimers are rather stabilized by the dipole–dipole interactions. Notably, the *n*-Bu group does not participate in any specific intermolecular contacts and appears to disrupt the overall crystal ordering. Finally, it should be noted that similar interactions and molecular motifs can be expected in doped films (aggregate states) and neat films, although the aforementioned halogen interaction is naturally excluded, as both films are prepared by vacuum deposition.

**Figure 2:**
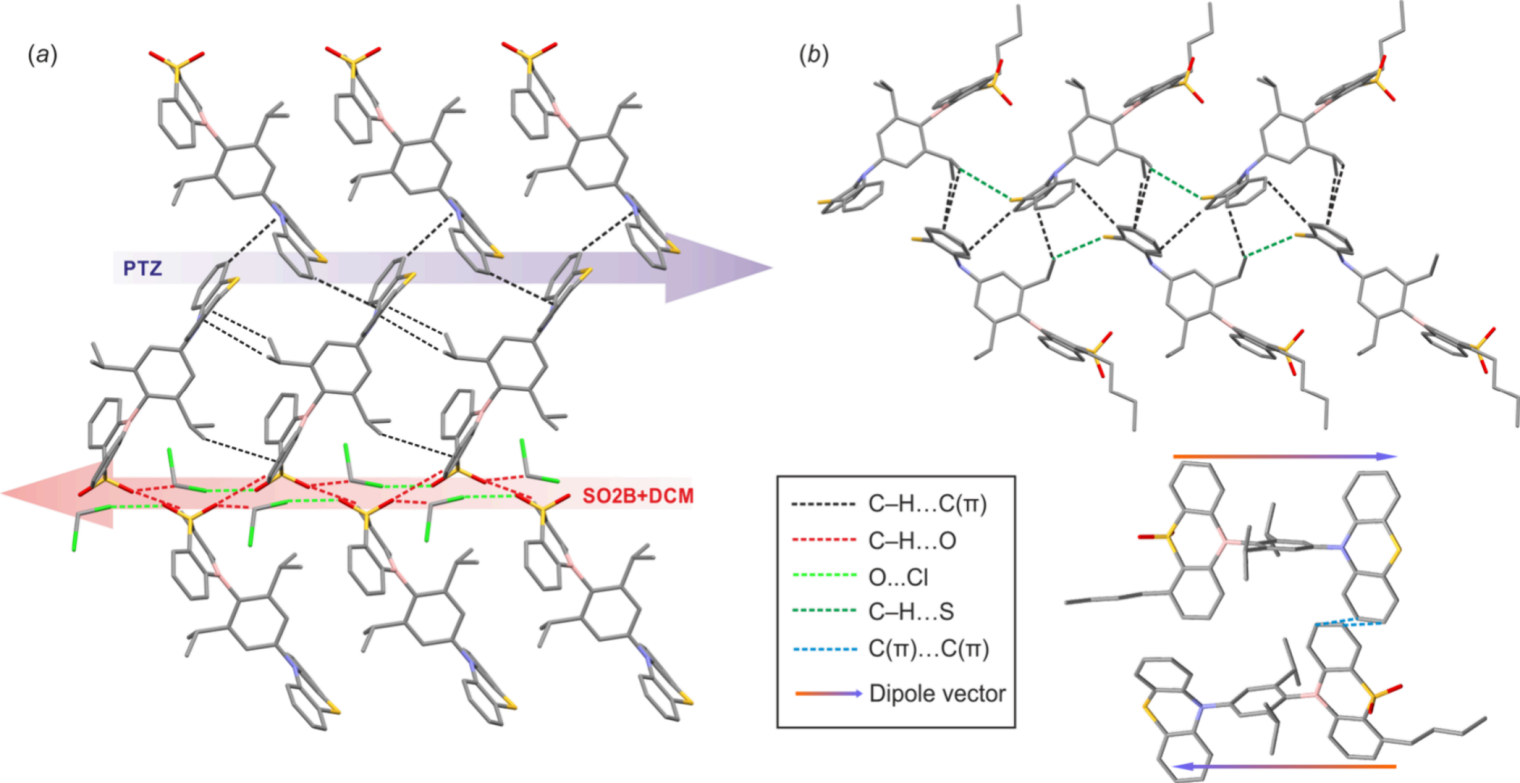
Main supramolecular motifs in the crystal structures of (a) **PTZ-Dipp-SO2B** and (b) **PTZ-Dipp-(Bu)­SO2B**.

### Photophysics

In order to gain insight into the potential differences between **PTZ-Dipp-SO2B** and **PTZ-Dipp-(Bu)­SO2B**, we studied their behavior in dilute solution and guest–host films as well as in neat films. Pertinent photoluminescence and absorption characteristics are summarized in Table 1. Overall, the two emitters behave similarly in dilute methylcyclohexane (MCH) solutions and zeonex films with the λ_em_ systematically slightly red-shifted in **PTZ-Dipp-SO2B** with respect to **PTZ-Dipp-(Bu)­SO2B** (Figure S2, SI). This slight red-shift results from the weak electron donating properties of the *n*-butyl group. The absorption spectra reveal an extremely weak S_0_ → S_1_ CT absorption, indicating in turn a very slow S_1_ → S_0_ luminescence transition. Both of these complexes display relatively long prompt fluorescence lifetimes, τ_PF_ ≈ 300–400 ns in methylcyclohexane (MCH) and τ_PF_ ≈ 600–700 ns in zeonex, with TADF lifetimes of τ_DF_ ≈ 4–5 μs in MCH and τ_DF_ ≈10–13 μs in zeonex. However, these molecules display high Φ_PL_ > 0.8 and *k*_r_^S^ ≈ 10^6^ s^–1^ comparable to *k*_r_ in some Ir­(III) complexes.^34^ The large τ_PF_ and the relatively small *k*_r_^S^ associated with a very small oscillator strength, *f*(S_1_ → S_0_) in these emitters are in line with the conclusions of our previous study.^31^ These figures agree with the same value obtained from the Strickler–Berg approach for the S_0_ → S_1_ CT transition, at *k*_r_^S^ = 1.1 × 10^6^ s^–1^ and 1.7 × 10^6^ s^–1^ for **PTZ-Dipp-SO2B** and **PTZ-Dipp-(Bu)­SO2B**, respectively. This behavior of the two emitters demonstrates a very efficient suppression of singlet nonradiative decay, allowing for this extremely slow prompt fluorescence decay with only very small energy losses. This extremely slow singlet decay results in a pronounced prompt fluorescence quenching by molecular oxygen, as seen in Figure S3. This case is a strong indication that simply comparing PL intensity in air-equilibrated and degassed solution cannot be used for quantitative assessments of TADF. We hypothesize that the very low nonradiative decay rate constant *k*_nr_ required to observe efficient emission from such a long-lived ^1^CT singlet state originates from the highly decoupled donor and acceptor orbitals and the B–C bridging bond, so the high frequency C–C and C=C vibrations cannot act as effective quenching modes.

**Table 1:** Summary of the Photophysical Characteristics of **PTZ-Dipp-SO2B** and **PTZ-Dipp-(Bu)­SO2B**

compound	solvent/matrix^a^	λ_abs_/nm (ε/10^3^ M^–1^ cm^–1^)^b^	λ_em_/nm^c^	Φ_PL_ ^d^	DF/PF ratio^e^	*k*_r_^S^/10^5^ s^–1^ ^f^	*k*_ISC_/10^5^ s^–1^ ^g^	*k*_rISC_/10^5^ s^–1^ ^h^	τ_**PF**_ **/ns** ^i^	τ_**DF**_ **/μs** ^j^
**PTZ-Dipp-SO2B**	MCH	299 (14.7), 438 (0.13)	577	0.88	1.5	8.5	15.6	2.8	415	4.6
	zeonex	-	588	0.93	0.5	9.1	5.7	0.4	672	13.1
	powder^k^	-	722	-	-	-	-	-	8 (25%); 28 (48%); 120 (27%)	-
	neat film^l^	-	706	0.01	-	-	-	-	5.6 (31%); 28.5 (69%)	-
**PTZ-Dipp-(Bu)SO2B**	MCH	297 (17.2), 431 (0.12)	561	0.78	1.9	9.3	24.6	2.6	295	5.6
	zeonex	-	570	1.0	0.8	9.2	7.1	0.8	614	10.3
	powder^k^	-	602	-	-	-	-	-	27 (16%); 116 (84%)	-
	neat film^l^	-	680	0.03	0.2	-	-	-	7.3 (16%); 56.8 (84%)	0.6 (74%); 2.2 (26%)

a MCH – methylcyclohexane, *c* = 10^–5^ M, zeonex, 1% w/w.

b Absorption maxima and the associated extinction coefficients.

c Emission maxima.

d Photoluminescence quantum yield.

e Ratio of delayed fluorescence to prompt fluorescence.

f Singlet radiative decay rate constant.

g Intersystem crossing rate constant.

h Reverse intersystem crossing constant.

i Prompt fluorescence lifetime.

j Delayed fluorescence lifetime.

k Sample not studied beyond steady state photoluminescence and TCSPC.

l *k*_r_^S^, *k*_ISC_, and *k*_rISC_ were not determined due to a relatively low Φ_PL_ and low DF/PF ratio or no DF detected at RT.

Our emitters display a significantly lower Φ_PL_ in powder (<0.1) and neat film than in solution, and a visibly red-shifted PL, at λ_em_ ≈ 600–700 nm with respect to their PL in methylcyclohexane. It is here where the differences between the two materials become more apparent as the *n*-butyl group affects the PL properties more significantly in the condensed phase. In both film and powder, the λ_em_ is closer to ∼700 nm for **PTZ-Dipp-SO2B** but closer to ∼600 nm for **PTZ-Dipp-(Bu)­SO2B**. The Φ_PL_ is 3-fold larger in **PTZ-Dipp-(Bu)­SO2B** than in the butyl-free counterpart, which cannot be explained simply with the energy gap law but rather by an aggregate-destabilizing effect of the appended alkyl chain. The significant PL red shifts of both **PTZ-Dipp-SO2B** and **PTZ-Dipp-(Bu)­SO2B** in neat films can be explained by aggregation. As seen in Figure S5, the absorption spectra of neat films feature an additional absorption band in the 400–600 nm range that is notably stronger than the CT absorption bands observed in solution within the 400–500 nm range. Such behavior is indicative of aggregation. The band in question is stronger in **PTZ-Dipp-SO2B** than in **PTZ-Dipp-(Bu)­SO2B**, suggesting that the butyl group partially disturbs aggregate formation, potentially yielding smaller aggregates. We may hypothesize that aggregation is also the cause of the red-shifted emission in powders.

The time-resolved PL spectra and decay traces in MCH, zeonex, the OLED host mCP:PO-T2T, and neat film are shown in detail in Figures S6, S8, S10, S11, S13, and S14. Selected data are listed in Figure 3. The characteristics of these two emitters are consistent with the typical behavior of D–A molecules displaying TADF, with occasional weak blue and short-lived local emission visible in the early times, followed by prompt CT fluorescence and TADF originating from the lowest ^1^CT state. We observe a pronounced contribution of the mCP:PO-T2T blue exciplex PL at early delay times when our SO2B emitters are studied in the OLED host. The TADF component displays a characteristic dependence on temperature. The prompt fluorescence and delayed PL spectra are identical at every temperature studied, suggesting that Δ*E*_ST_ is likely to be very small. We use the temperature dependence of TADF to estimate the Δ*E*_ST_ (Figures S15 and S16), with a value of 49 ± 2 meV obtained for **PTZ-Dipp-SO2B** and 49 ± 1 meV for **PTZ-Dipp-(Bu)­SO2B**. This experimental value suggests that although the S_1_–T_1_ energy gap is very small, there still exists thermal activation for TADF due to the coupling with the T_n_ state to enable rISC. We also confirm the intramolecular nature of the delayed fluorescence with a linear power dependence (Figures S7, S9, S12, and S15).

**Figure 3:**
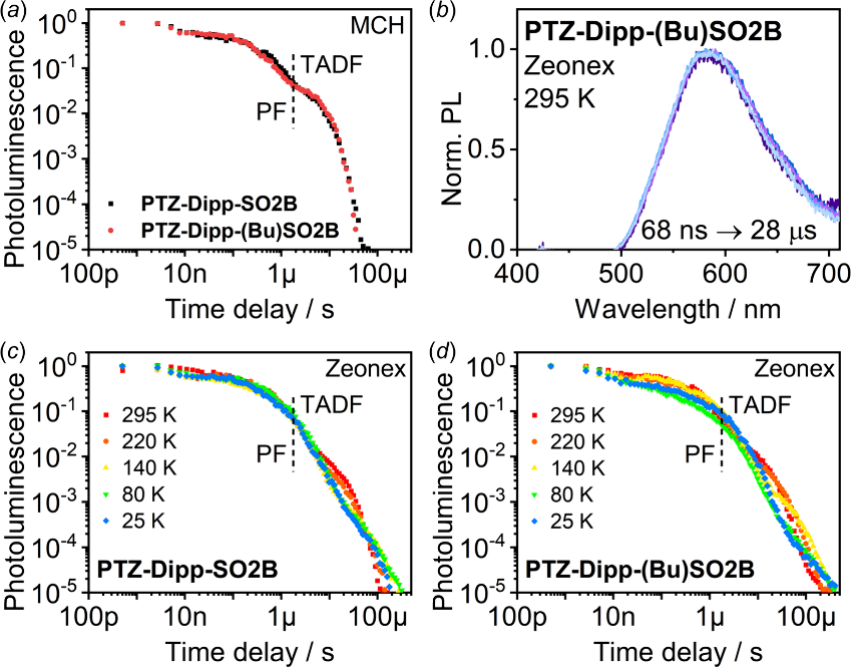
Time-resolved photoluminescence study: (a) photoluminescence decay of **PTZ-Dipp-SO2B** and **PTZ-Dipp-(Bu)­SO2B** in methylcyclohexane (MCH) at RT, *c* = 10^–5^ M; (b) time-resolved PL spectra of **PTZ-Dipp-(Bu)­SO2B** in zeonex (1% w/w) at RT; (c, d) photoluminescence decay of **PTZ-Dipp-SO2B** and **PTZ-Dipp-(Bu)­SO2B** in zeonex (1% w/w) at temperatures indicated in the respective figure legends.

We do not observe a TADF component in the neat film of **PTZ-Dipp-SO2B**, likely due to its very low PLQY caused by the nonradiative decay. At lower temperature where the nonradiative processes are suppressed, a longer-lived signal is present, which we believe to be TADF. However, a TADF contribution in the neat film of **PTZ-Dipp-(Bu)­SO2B** can clearly be seen even at 295 K, which agrees with its higher Φ_PL_. The TADF component increases, rather than decreasing, upon cooling—a sign that the effects of TADF quenching at lower temperatures are less significant than suppression of the nonradiative decay. This would be the case when the nonradiative decay dominates at RT, while Δ*E*_ST_ ≈ 0.

### Electrochemistry

Both molecules display reversible first oxidation and reduction waves as well as an irreversible second oxidation wave (Figure S18). The first oxidation wave is associated with the phenothiazine donor, while the reduction wave with the SO2B acceptor. Both molecules display identical oxidation half-wave potentials at *E*_1/2_^ox1^ = 0.27 V and *E*_1/2_^ox2^ = 0.89 V vs Fc/Fc^+^ (Table S4). The two molecules differ in the reduction half-wave potential: *E*_1/2_^red^ = −1.79 V for **PTZ-Dipp-(Bu)­SO2B** and *E*_1/2_^red^ = −1.70 V for **PTZ-Dipp-SO2B**, resulting from the σ-donor properties of the *n*-butyl group.

### Computations

TD-DFT calculations at the CAM-B3LYP/6-311++G­(d,p) level of theory show that both compounds possess very similar electronic structures. The energy of HOMO, localized at the phenothiazine moiety, equals to −5.27 eV, while LUMO (situated at SO2B) has an energy of −2.99 eV for **PTZ-Dipp-SO2B** and increases to −2.87 eV for **PTZ-Dipp-(Bu)­SO2B,** reflecting the electron-donating properties of the *n*-butyl group. The pronounced separation of the frontier orbitals indicates the strong CT character of the excited singlet (S_1_) and triplet states (T_1_) and a small Δ*E*_ST_ below 0.05 eV (Figure 4). This is consistent with a negligible *f*(T_1_ → S_0_) and large τ_PF_. Moreover, the identical symmetry of the natural transition orbitals (NTOs) leads to negligible spin–orbit coupling (SOC) values (<0.01 cm^–1^). This suggests that the rISC is likely mediated *via* vibronic coupling with the T_2_ state,^35^ which is also of the CT nature. The calculated S_1_–T_2_ SOC values for **PTZ-Dipp-SO2B** and **PTZ-Dipp-(Bu)­SO2B** are 2.64 cm^–1^ and 2.98 cm^–1^, respectively, supporting the S_1_–T_2_ spin vibronic coupling mechanism, which is consistent with the similar mechanism operating in the carbazole analogue (**CZ-Dipp-SO2B**) as reported in our previous study.^31^ Finally, it should be noted that while DFT calculations correctly predict small S–T energy separation, orbital nature, and spin–orbit couplings, the absolute singlet and triplet CT state energy values are underestimated. This leads to larger separation of the T_1_–T_2_ gap,^36,37^ while it is believed that the experimental value of ∼0.05 eV reflects the experimental gap as studied for carbazole-based systems.

**Figure 4:**
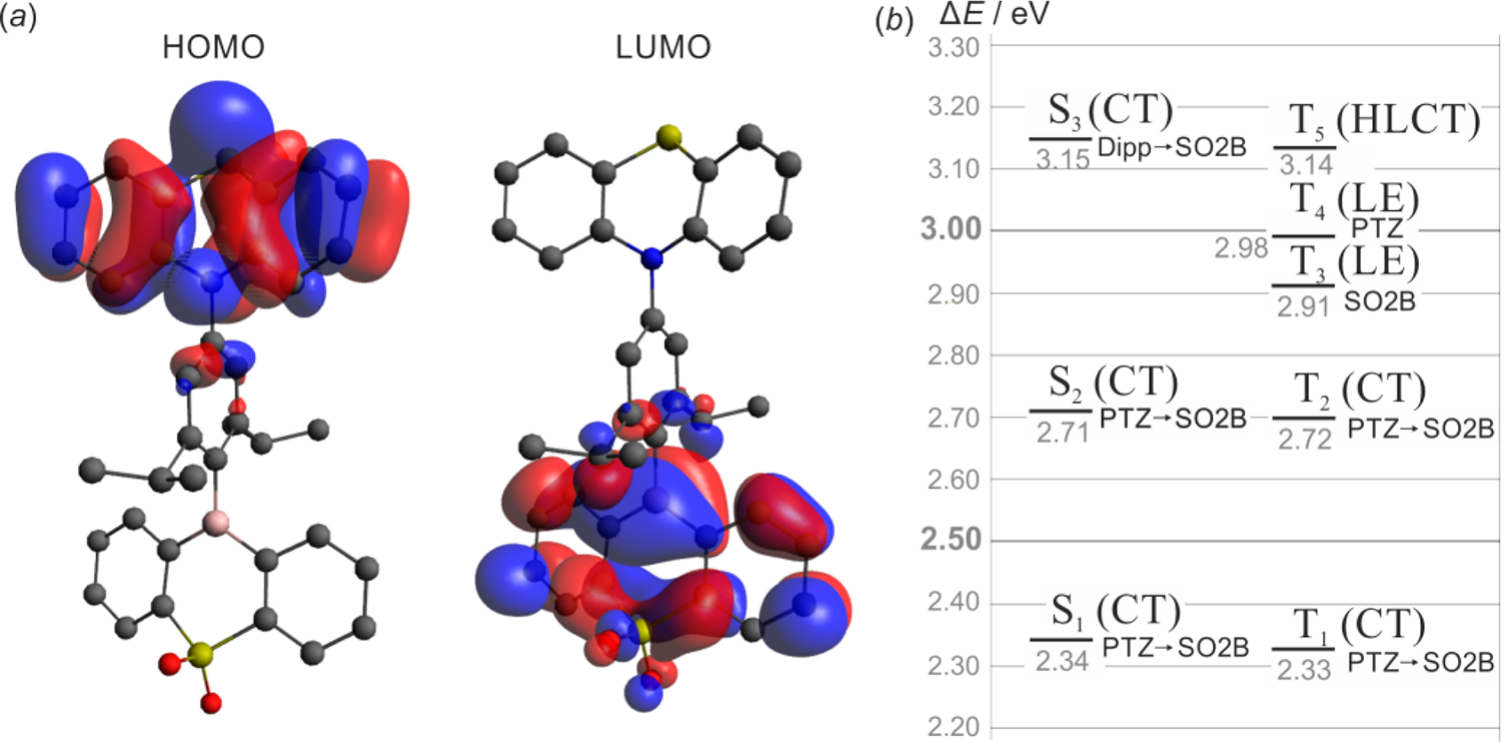
(a) Frontier molecular orbital contours (0.02 au) and (b) excited state energy diagrams for **PTZ-Dipp-SO2B**. The indices used in state labeling indicate the direction of the energy transfer, for CT states, and localization of the state for LE states. The results for **PTZ-Dipp-(Bu)­SO2B** are comparable and are shown in the Supporting Information (Figure S21).

### Electroluminescence

Following the high performance of the OLEDs featuring similar emitters reported in our earlier work,^31^ we decided to use the same OLED structure in our study. We have found that slightly lowering the doping concentration of the emitter to 7%, rather than 10% as in the original study, provides a higher performance, and we use this approach in this study. Devices **Dev 1** and **Dev 2** using, respectively, **PTZ-Dipp-SO2B** or **PTZ-Dipp-(Bu)­SO2B** feature an OLED architecture: ITO | HAT-CN (10 nm) | TSBPA (40 nm) | mCP (2 nm) | mCP:PO-T2T (80:20) co 7% **PTZ-Dipp-SO2B** or **PTZ-Dipp-(Bu)­SO2B** (20 nm) | PO-T2T (50 nm) | LiF­(0.8 nm) | Al (100 nm). We have also produced nondoped OLEDs **Dev 3** and **Dev 4** featuring neat films of **PTZ-Dipp-SO2B** and **PTZ-Dipp-(Bu)­SO2B**, respectively, using the structure: ITO | HAT-CN (10 nm) | TSBPA (40 nm) | **PTZ-Dipp-SO2B** or **PTZ-Dipp-(Bu)­SO2B** (5 nm) | PO-T2T (50 nm) | LiF­(0.8 nm) | Al (100 nm). The results are presented in Figure 5 and Figures S25–S30 in the SI, while the associated numerical characteristics are presented in Table S6. The turn-on voltage of the OLEDs featuring **PTZ-Dipp-(Bu)­SO2B** is lower than that of those using **PTZ-Dipp-SO2B**. This can be rationalized with the difference in LUMO energy, which at −3.38 eV is closer to that of the electron-transport layer PO-T2T at ca. –3.1–3.2 eV than in the case of **PTZ-Dipp-SO2B**, at −3.48 eV. The OLEDs highlight the principal differences between the two emitters, which are not immediately visible in a solution, resulting in visibly different EL wavelengths. **PTZ-Dipp-SO2B** displays a systematically more red-shifted emission than the **PTZ-Dipp-(Bu)­SO2B** in all studied OLEDs. The doped OLEDs **Dev 1** (**PTZ-Dipp-SO2B**) and **Dev 2** (**PTZ-Dipp-(Bu)­SO2B**) display orange-red EL, λ_EL_ = 616 nm and λ_EL_ = 601 nm, respectively, while the nondoped devices **Dev 3** and **Dev 4** show deep red to NIR electroluminescence, λ_EL_ = 709 nm and λ_EL_ = 681 nm, respectively. The OLEDs are systematically more efficient for **PTZ-Dipp-(Bu)­SO2B** than for **PTZ-Dipp-SO2B** by a factor of 2–3 (Figures S25 and S26), which is attributed to the higher solid state Φ_PL_ of the butylated counterpart. OLEDs **Dev 1** and **Dev 2** display maximum EQEs of 5.4 and 12.2%, respectively, while their maximum luminance achieves 3300 and 15300 cd m^–2^, respectively (Figure S27). Likewise, the corresponding OLEDs **Dev 3** and **Dev 4** display a maximum EQE of 0.4 and 1.0%, respectively, while their maximum radiant emittance achieves 0.40 and 0.63 mW cm^–2^, respectively. Devices **1**–**4** display a noticeable efficiency roll-off, which can be quantified using the critical current density at 90% EQE_max_, *J*_90%_, a metric introduced by Murawski et al.^38^ The *J*_90%_ values of the OLEDs are 0.4 mA cm^–2^ (**1**), 0.2 mA cm^–2^ (**2**), 4.8 mA cm^–2^ (**3**), and 2.4 mA cm^–2^ (**4**). While values of 1–10 are common among both TADF and phosphorescent emitters, *J*_90%_ < 1 mA cm^–2^ indicates severe roll-off. We hypothesize that this particularly severe roll-off may be due to the shifting of the center of the recombination zone toward the mCP | EML interface at higher current densities. We believe that the significant divergence in EQE between **Dev 1** and **Dev 2** as well as **Dev 3** and **Dev 4** cannot simply be explained with the energy gap law as the differences in EL wavelength are small. We also note that the difference persists among all studied devices, as evidenced by comparison of the average EQE values. We believe that the inclusion of the pendant alkyl to the SO2B unit not only slightly alters the electron density on the same molecule but also alters the average polarity of the surrounding environment. The pendant butyl chain acts somewhat as a nonpolar solvent molecule in this sense that it is rather free to move around as if a relatively unrestricted molecule would do. We are unsure to the exact nature of this effect—it may be associated with both the change of the average polarity/polarizability around the molecule and the disruption of packing between stacked molecules in film. Accordingly, the **PTZ-Dipp-SO2B** displays redder PL resultant of a higher polar environment and aggregation, while the butyl group in **PTZ-Dipp-(Bu)­SO2B** acts as an intercalator, disturbing the intermolecular π···π interactions and hence reducing luminescence quenching. The use of D–A TADF emitters in neat films is one of the main strategies to achieve NIR OLEDs.^15^ Although our systems produce rather modest efficiencies, they pose as an important proof-of-concept example for the use of linear alkyl chains to improve the NIR/red OLED efficiency.

**Figure 5:**
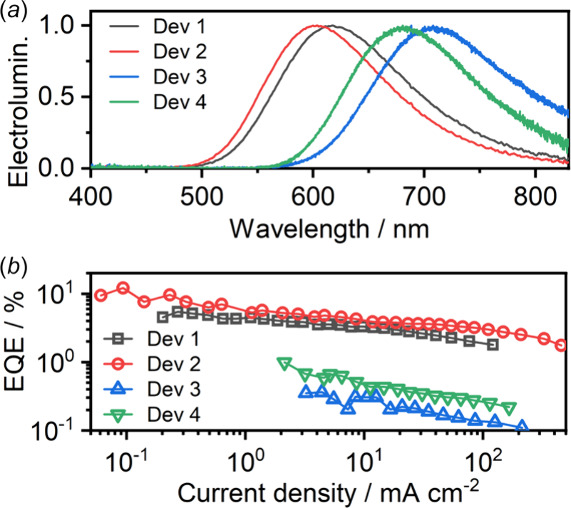
Characteristics of the OLED devices **Dev 1** to **Dev 4**: (a) electroluminescence spectra; (b) external quantum efficiency (EQE) vs current density.

## Conclusions

To summarize, we have presented two new and structurally similar emitters, namely, **PTZ-Dipp-SO2B** and **PTZ-Dipp-(Bu)­SO2B**. They differ in such a way that the latter contains a pendant *n*-butyl group attached to the SO2B acceptor. As we find that their photophysical behaviors in solution and in high dilution in film are alike, they display significant differences in PL efficiency at higher concentrations and in neat films. The pendant *n*-butyl group acts as a weak σ-donor, slightly weakening the SO2B acceptor, but without an important effect on TADF properties and Φ_PL_ in dilution. The alkyl group plays an important role by lowering the effective polarity/polarizability in the solid state and disrupting the intermolecular π···π interactions. Since this effect is similar to that observed earlier in solvate crystals containing hexane, we propose to name it the internal solvation effect. The advantage of the internal solvation is that the solvent-like fragment is chemically bound to the emitter, unlike volatile solvent molecules, which are relatively free. Hence, the beneficial effect of controlling solvation in the solid state can be easily reproduced in, for example, neat amorphous layers and through thermal evaporation. As a result, this effect is going to be especially important in nondoped NIR OLEDs, where fine control over intermolecular interactions is key to high EQE.

## Experimental Section

### General

Starting materials including *n*-BuLi (2.5 M solution in hexane), BCl_3_ (1 M solution in hexane), phenothiazine, CuI, and K_2_CO_3_ were obtained as received without further purification. Dry Et_2_O under molecular sieves was obtained from Acros. Hexane, THF, and DCM were purified using MBraun SPS and stored under argon. Reactions and manipulations involving air and moisture-sensitive reagents were carried out under an argon atmosphere. 10-Hydroxy-10*H*-dibenzo­[*b*,*e*]­[1,4]­thiaborinine 5,5-dioxide and 2-bromo-5-iodo-1,3-diisopropylbenzene were available from our previous studies.^31^

^1^H, ^11^B, and ^13^C NMR spectra were recorded on Agilent NMR 400 MHz and JEOL JNM-ECZL 600 MHz spectrometers. ^1^H and ^13^C chemical shifts were referenced to TMS by using known chemical shifts of solvent residual peaks. ^11^B NMR chemical shifts are given relative to BF_3_·Et_2_O. In the ^13^C NMR spectra, the resonances of boron-bound carbon atoms were not observed as a result of their broadening by the quadrupolar boron nucleus. In addition, signals corresponding to carbon atoms bonded to the SO_2_ group show low intensities on ^13^C NMR spectra. High resolution MS spectra were recorded using a MALDI SYNAPT G2-S HDMS (Waters) coupled with an ACQUITY UPLC I-Class System (Waters) and magnetic sector mass spectrometer AutoSpec Premier (Waters, USA), equipped with an electron impact (EI) ion source and the EBE double focusing geometry mass analyzer. The HRMS of **1** and **2** were not measured due to their fast hydrolysis to 10-hydroxy-10*H*-dibenzo­[*b*,*e*]­[1,4]­thiaborinine 5,5-dioxide under standard analytical conditions.

### Synthesis

#### 10-Chloro-10*H*-dibenzo­[*b*,*e*]­[1,4]­thiaborinine 5,5-Dioxide (**1**)

10-Hydroxy-10*H*-dibenzo­[*b*,*e*]­[1,4]­thiaborinine 5,5-dioxide^31^ (0.17 g, 0.70 mmol) was placed in a 50 mL Schlenk flask and suspended in 5 mL of dry DCM. Then, the mixture was cooled to −78 °C in a dry ice/acetone bath, and 1 M solution of BCl_3_ in hexane (0.7 mL, 0.7 mmol) was added with a syringe. The reaction mixture became clear during the addition of BCl_3_, and the product was precipitated from the solution. The mixture was allowed to warm up slowly and stirred overnight. DCM was decanted, and then, 40 mL of dry DCM was added to the remaining solid. After stirring for ca. 30 min, the product dissolved in DCM, while the inorganic boron residue remained as an insoluble material. The mixture was filtered under an argon atmosphere, and the clear filtrate was evaporated to dryness under reduced pressure to give **1** as a white solid (0.17 g, 93%). ^1^H NMR (400 MHz, CDCl_3_) δ = 8.29 (ddd, *J* = 7.8, 1.1, 0.6 Hz, 2H), 7.86–7.79 (m, 4H), 7.63 (td, *J* = 7.4, 1.1 Hz, 2H) ppm. NMR analysis is consistent with the reported data for this compound.^31^ The obtained product was used for the subsequent reaction on the same day. Note that **1** is moisture sensitive and should be stored under an inert atmosphere.

#### 10-Isopropoxy-10*H*-dibenzo­[*b*,*e*]­[1,4]­thiaborinine 5,5-Dioxide (**2**)

Dry propan-2-ol (0.22 mL, 2.9 mmol) was added to a suspension of **1** (0.17, 0.65 mmol) in 30 mL of dry DCM at 0 °C. The mixture was gradually warmed to room temperature and stirred for 1.5 h. Volatiles were evaporated, and dry hexane (5 mL) was added to the remaining oily residue. Volatiles were evaporated again to give the product as an off-white solid. Yield 0.19 g (95%). The compound was used in the subsequent reaction step without additional purification. ^1^H NMR (400 MHz, CDCl_3_) δ 8.19–8.12 (m, 2H), 7.89–7.85 (m, 2H), 7.60 (m, 4H), 5.26 (br, 1H), 1.48 (b, 6H) ppm.

#### 10-(4-Bromo-3,5-diisopropylphenyl)-10*H*-phenothiazine (**3**)

Under an argon atmosphere, a mixture of 2-bromo-5-iodo-1,3-diisopropylbenzene (1.84 g, 5.0 mmol), 10*H-*phenothiazine (1.00 g, 5.0 mmol), CuI (0.10 g, 0.5 mmol), and anhydrous K_2_CO_3_ (1.40 g, 10 mmol), and dry DMF (10 mL) was stirred for 36 h at 170 °C. The reaction mixture was diluted with CH_2_Cl_2_ (40 mL) and washed with water (2 × 15 mL). The aqueous phase was extracted with CH_2_Cl_2_ (20 mL), and the combined organic extracts were dried over MgSO_4_ and filtered. Purification with column chromatography (silica gel with 5% ethyl acetate in hexane, *t*_r_ = 0.5) afforded **3** as a white solid. Yield 1.75 g (80%). ^1^H NMR (600 MHz, CDCl_3_) δ 7.15 (s, 2H), 7.03 (dd, *J* = 7.4, 1.7 Hz, 2H), 6.88–6.78 (m, 4H), 6.16 (d, *J* = 8.1 Hz, 2H), 3.57 (hept, *J* = 6.9 Hz, 2H), 1.26 (d, *J* = 6.9 Hz, 12H) ppm. ^13^C­{^1^H} NMR (151 MHz, CDCl_3_) δ: 151.0, 144.4, 140.3, 127.1, 126.9, 126.8, 126.0, 122.6, 120.1, 115.8, 33.9, 23.1 ppm. ^1^H NMR (600 MHz, C_6_D_6_) δ = 7.06 (s, 2H), 6.97 (dd, *J* = 7.6, 1.6 Hz, 2H), 6.67 (ddd, *J* = 8.4, 7.3, 1.6 Hz, 2H), 6.60 (td, *J* = 7.4, 1.3 Hz, 2H), 6.27 (dd, *J* = 8.3, 1.2 Hz, 2H), 3.53 (hept, *J* = 6.8 Hz, 2H), 1.01 (d, *J* = 6.9 Hz, 12H) ppm. ^13^C­{^1^H} NMR (151 MHz, C_6_D_6_) δ: 151.4, 145.0, 140.9, 128.3, 127.3, 127.2, 126.2, 123.1, 120.8, 116.1, 34.1, 22.8 ppm. The ^1^H and ^13^C NMR analyses of compound **3** indicate some dynamic behavior within the phenothiazine ring. In turn, the NMR spectra in deuterated benzene give sharp resonances. NMR analysis is consistent with the reported data for this compound.^39^

#### 10-(2,6-Diisopropyl-4-(10*H*-phenothiazin-10-yl)­phenyl)-10*H*-dibenzo­[*b*,*e*]­[1,4]­thiaborinine 5,5-Dioxide (**PTZ-Dipp-SO2B**) and 4-Butyl-10-(2,6-diisopropyl-4-(10*H*-phenothiazin-10-yl)­phenyl)-10*H*-dibenzo­[*b*,*e*]­[1,4]­thiaborinine 5,5-Dioxide (**PTZ-Dipp-(Bu)­SO2B**)

*n*-BuLi (2.5 M in hexane, 0.27 mL, 0.68 mmol) was added to a solution of **3** (0.30 g, 0.68 mmol) in THF (5 mL) at −78 °C. After 1.5 h of stirring, a solution of **2** (0.19 g, 0.66 mmol) in THF (3 mL) was added with a syringe. The mixture was gradually warmed to room temperature and stirred overnight. It was quenched with HCl (2 M solution in Et_2_O, 0.4 mL). Volatiles were evaporated, and the oily residue was suspended in 15 mL of DCM. The mixture was extracted with water (3 × 15 mL) to remove the insoluble residue from the organic phase. Combined water phases were extracted with DCM (15 mL). Then, the combined organic phases were dried over MgSO_4_ and filtered. Solvents were evaporated, and the residue was subjected to separation by column chromatography on silica gel with DCM to give **PTZ-Dipp-SO2B** (0.108 g, 28%) and **PTZ-Dipp-(Bu)­SO2B** (0.102 g, 24%) as orange solids.

##### PTZ-Dipp-SO2B

Mp 240–242 °C; ^1^H NMR (600 MHz, CDCl_3_) δ 8.33 (d, *J* = 7.8 Hz, 2H, SO2B), 7.88–7.84 (t, *J* = 7.9 Hz, 2H, SO2B), 7.78 (d, *J* = 6.7 Hz, 2H, SO2B), 7.71 (t, *J* = 7.4 Hz, 2H, SO2B), 7.26 (s, 2H, Dipp), 7.08 (d, *J* = 7.6 Hz, 2H, PTZ), 7.01–6.92 (m, 2H, PTZ), 6.87 (t, *J* = 7.4 Hz, 2H, PTZ), 6.34 (d, *J* = 8.2 Hz, 2H, PTZ), 2.17 (hept, *J* = 6.6 Hz, 2H, *i*-Pr), 1.06 (d, *J* = 6.6 Hz, 12H *i*-Pr) ppm. ^13^C NMR (151 MHz, CDCl_3_) δ: 153.2, 146.7, 144.8, 142.2, 139.5, 135.6, 132.4, 127.1, 127.1, 125.0, 124.0, 122.6, 120.5, 115.9, 36.3, 24.2 ppm. ^11^B NMR (160.5 MHz, CDCl_3_, *T* = 55 °C, *c* = 0.055 M, 5000 scans) δ 63.4 ppm. HRMS (ESI, positive ion mode) Calcd. for C_36_H_33_BNO_2_S_2_^+^ [MH^+^]: 586.20403; found: 586.20374.

##### PTZ-Dipp-(Bu)­SO2B

Mp 213–215 °C; ^1^H NMR (400 MHz, CDCl_3_) δ 8.31 (d, *J* = 7.8 Hz, 1H, SO2B), 7.90 (ddd, *J* = 7.9, 6.3, 2.5 Hz, 1H, SO2B), 7.73–7.63 (m, 4H, SO2B), 7.58 (t, *J* = 7.5 Hz, 1H, SO2B), 7.24 (s, 2H, Dipp), 7.07 (dd, *J* = 7.5, 1.6 Hz, 2H, PTZ), 6.96 (ddd, *J* = 8.2, 7.3, 1.6 Hz, 2H, PTZ), 6.86 (td, *J* = 7.4, 1.3 Hz, 2H, PTZ), 6.34 (dd, *J* = 8.2, 1.2 Hz, 2H, PTZ), 3.42–3.34 (m, 2H, CH_2_CH_2_CH_2_CH_3_), 2.30 (hept, *J* = 6.6 Hz, 2H, *i*-Pr), 1.92–1.79 (m, 2H, CH_2_ CH_2_CH_2_CH_3_), 1.64–1.56 (m, 2H, CH_2_CH_2_ CH_2_CH_3_), 1.06–0.99 (m, 15H, *i*-Pr and CH_2_CH_2_CH_2_ CH_3_) ppm. ^13^C NMR (101 MHz, CDCl_3_) δ 152.9, 149.0, 144.7, 143.9, 142.3, 141.8, 138.8, 138.6, 138.2, 135.8, 131.9, 131.5, 126.9, 126.9, 124.8, 123.7, 122.4, 120.2, 115.7, 36.0, 33.9, 33.0, 24.0, 23.1, 14.1 ppm. ^11^B NMR (160.5 MHz, CDCl_3_, *T* = 55 °C, *c* = 0.055 M, 5000 scans) δ 62.4 ppm. HRMS (ESI, positive ion mode) Calcd. for C_40_H_40_BNO_2_S_2_^+^ [MH^+^]: 641.25880; found: 641.25747.

In the ^1^H NMR spectrum of **PTZ-Dipp-SO2B** the C_ar_–H (Dipp) resonance overlaps with the signal of the residual CHCl_3_ solvent. In the ^13^C­{^1^H}­NMR spectra, the resonances of boron-bound carbon atoms were not observed due to their broadening by the quadrupolar boron nucleus. The ^11^B NMR analysis performed in CDCl_3_ solution at ambient temperature shows the expected signal of the boron atom at about 62 ppm. It appears next to a very broad signal of the borosillicate glass, and its intensity is very low despite a rather high sample concentration (*c* = 0.055 M, i.e., 25 mg·mL^–1^) and prolonged acquisition time. The intensity of the signal increased at higher temperatures. Similar behavior was observed for SO2B-based TADFs studied by us previously.^31^

### Crystal Structure Determination

Single crystals of **PTZ-Dipp-SO2B** and **PTZ-Dipp-(Bu)­SO2B** were prepared by slow evaporation of their DCM solutions. The obtained single crystals were measured using an X-ray diffractometer according to the published procedure.^31^ Crystallographic data are listed in Table S1. CCDC deposition numbers: 2469443 (**PTZ-Dipp-SO2B**), 2469444 (**PTZ-Dipp-(Bu)­SO2B**).

### Electrochemistry

Cyclic voltammetry experiments were conducted following the same procedure as described in our earlier works.^40,41^ The ionization potential (IP) and electron affinity (EA) were calculated using the expressions described earlier:^42,43^ IP = *E*_ox_^CV^ + 5.1 [eV]; EA = E_red_^CV^ + 5.1 [eV].

### Photophysics

UV–vis absorption spectra were recorded by using a Hitachi UV-2300II spectrometer. The emission spectra of solutions were recorded using an Edinburgh FS5 equipped with an enhanced range photomultiplier detector (PMT-EXT). The measurements were performed at room temperature, according to published procedures.^44,45^ Suprasil quartz cuvettes (10 mm) were used; 1.5 nm slit widths were used for absorption and emission spectra. To eliminate any background emission, the spectrum of pure solvent was subtracted from the recorded spectra. Photoluminescence quantum yields were determined in diluted solutions (*A* ≈ 0.03 at the excitation wavelength used) by comparison with known standards – quinine sulfate in 0.1 M H_2_SO_4_ (QY = 0.546). The concentrations of all samples were adjusted to reach absorbance similar to the absorbance of the reference solution at the excitation wavelength.

Details on basic optical spectroscopy experiments as well as time-resolved experiments involving time-correlated single photon counting (TCSPC), a sensitive gated iCCD camera, and temperature-dependent studies have been described elsewhere.^31^ Further details on the transient photoluminescence setup are available elsewhere.^46^

### Electroluminescent Devices

OLEDs were fabricated using the Kurt J. Lesker vacuum deposition system and following procedures described earlier by some of the current authors.^31,47^ Additional details on device fabrication can be found elsewhere.^48^

Listed below are the materials used for OLED fabrication, their origin and quality/purity grate, and their role in the device: HAT-CN (hole injection material) – dipyrazino­[2,3-f:2’,3′-h]­quinoxaline-2,3,6,7,10,11-hexacarbonitrile (sublimed, LUMTEC); TSBPA (hole transport material) – 4,4’-(diphenylsilanediyl)­bis­(*N*,*N*-diphenylaniline) (LUMTEC); mCP (hole transport and cohost material) – 1,3-bis­(carbazol-9-yl)­benzene (sublimed, LUMTEC); PO-T2T (electron transport and cohost material) – 2,4,6-tris­[3-(diphenylphosphinyl)­phenyl]-1,3,5-triazine (LUMTEC); LiF (electron injection material, 99.995%, Sigma-Aldrich); Al (cathode material, 99.9995%, Lesker).

### Thermal Characterization

Thermogravimetric analyses were performed on a TGA/DSC1 (Mettler-Toledo) system under a continuous flow of argon at *T* = 30–450 °C with a ramp rate of 10 K·min^–1^. Samples of 3.8 mg (**PTZ-Dipp-SO2B**) and 4.2 mg (**PTZ-Dipp-(Bu)­SO2B**) were prepared in covered ceramic crucibles. An empty crucible was used as a reference, and α-Al_2_O_3_ was used for instrument calibration. Melting points (*T*_m_) were determined from visual observations with an automated Mettler Toledo DP70 Melting Point system. The obtained values correspond to the melting points derived from DSC thermograms.

### Quantum Chemical Calculations

Quantum chemical calculations were performed using the *Gaussian16* program package.^49^ In the first step, the molecules were optimized at the B3LYP^50–52^ /6-311++G­(d,p)^53^ level of theory. Following geometry optimization, vibrational frequencies were calculated, confirming the stability of the optimized structures (no imaginary frequencies). Excited singlet and triplet state geometries were obtained with TD-DFT methods at the CAM-B3LYP^54^/6-311++G­(d,p) level of theory. The calculations show that the molecular geometries of singlet and triplet excited states are generally preserved from the corresponding ground states, with the exception of the phenothiazine unit, which adopts either bent (S_0_, S_2_, and T_2_) or flat (S_1_ and T_1_) geometry. In order to determine the nature of singlet and triplet excited states, natural transition orbitals^55^ were calculated and visualized with the *Avogadro* program (Figures S22, S23).^56^ The SOCME values were calculated with ORCA at the same level of theory.^57^

## Supplementary Material



## Data Availability

A version of this manuscript has been submitted to ChemRxiv: https://doi.org/10.26434/chemrxiv-2025-jq01l. Our supporting research data is available from Zenodo: https://doi.org/10.5281/zenodo.18163380.

## Notes

Synthetic procedures, ^1^H, ^13^C­{^1^H}, ^1^H–^1^H COSY, and ^1^H–^13^C HSQC NMR spectra, HRMS, experimental details, details on X-ray crystallography, TGA, supplementary electrochemistry, photophysics, computational, and OLED characterization results (PDF)

## References

[1] Vasilopoulou M., Fakharuddin A., García de Arquer F. P., Georgiadou D. G., Kim H., Mohd Yusoff A. R. B., Gao F., Nazeeruddin M. K., Bolink H. J., Sargent E. H. (2021). Advances
in solution-processed near-infrared light-emitting diodes. Nat. Photonics.

[2] “New OLED gadget: Oppo F21 Pro/F21 Pro 5G,” can be found underhttps://www.oled-info.com/oppo-f21-pro-f21-pro-5g.

[3] Kamtekar K. T., Monkman A. P., Bryce M. R. (2010). Recent Advances
in White Organic
Light-Emitting Materials and Devices (WOLEDs). Adv. Mater..

[4] Fahlteich J., Steiner C., Top M., Wynands D., Wanski T., Mogck S., Kucukpinar E., Amberg-Schwab S., Boeffel C., Schiller N. (2015). Roll-to-roll manufacturing of functional
substrates and encapsulation films for organic electronics: technologies
and challenges. SID Symp. Dig. Technol. Pap..

[5] Xu R.-P., Li Y.-Q., Tang J.-X. (2016). Recent
advances in flexible organic
light-emitting diodes. J. Mater. Chem. C.

[6] Wei Y.-C., Wang S. F., Hu Y., Liao L.-S., Chen D.-G., Chang K.-H., Wang C.-W., Liu S.-H., Chan W.-H., Liao J.-L., Hung W.-Y., Wang T.-H., Chen P.-T., Hsu H.-F., Chi Y., Chou P.-T. (2020). Overcoming the energy
gap law in near-infrared OLEDs by exciton–vibration decoupling. Nat. Photonics.

[7] Pander P., Daniels R., Zaytsev A. V., Horn A., Sil A., Penfold T. J., Williams J. A. G., Kozhevnikov V. N., Dias F. B. (2021). Exceptionally fast radiative decay of a dinuclear platinum
complex through thermally activated delayed fluorescence. Chem. Sci..

[8] Tuong
Ly K., Chen-Cheng R. W., Lin H. W., Shiau Y. J., Liu S. H., Chou P. T., Tsao C. S., Huang Y. C., Chi Y. (2017). Near-infrared organic light-emitting diodes with very high external
quantum efficiency and radiance. Nat. Photonics.

[9] Minaev B., Baryshnikov G., Agren H. (2014). Principles of phosphorescent
organic
light emitting devices. Phys. Chem. Chem. Phys..

[10] Uoyama H., Goushi K., Shizu K., Nomura H., Adachi C. (2012). Highly efficient
organic light-emitting diodes from delayed fluorescence. Nature.

[11] Cho Y. J., Yook K. S., Lee J. Y. (2014). A universal host material for high
external quantum efficiency close to 25% and long lifetime in green
fluorescent and phosphorescent OLEDs. Adv. Mater..

[12] Kim B. S., Lee J. Y. (2014). Engineering of mixed
host for high external quantum
efficiency above 25% in green thermally activated delayed fluorescence
device. Adv. Funct. Mater..

[13] Zeng W., Lai H.-Y., Lee W.-K., Jiao M., Shiu Y.-J., Zhong C., Gong S., Zhou T., Xie G., Sarma M., Wong K.-T., Wu C.-C., Yang C. (2018). Achieving
nearly 30% external quantum efficiency for orange–red organic
light emitting diodes by employing thermally activated delayed fluorescence
emitters composed of 1,8-naphthalimide-acridine hybrids. Adv. Mater..

[14] Dos
Santos J. M., Hall D., Basumatary B., Bryden M., Chen D., Choudhary P., Comerford T., Crovini E., Danos A., De J., Diesing S., Fatahi M., Griffin M., Gupta A. K., Hafeez H., Hämmerling L., Hanover E., Haug J., Heil T., Karthik D., Kumar S., Lee O., Li H., Lucas F., Mackenzie C. F. R., Mariko A., Matulaitis T., Millward F., Olivier Y., Qi Q., Samuel I. D. W., Sharma N., Si C., Spierling L., Sudhakar P., Sun D., Tankelevičiu̅tė E., Duarte Tonet M., Wang J., Wang T., Wu S., Xu Y., Zhang L., Zysman-Colman E. (2024). The golden age of thermally activated
delayed fluorescence materials: design and exploitation. Chem. Rev..

[15] dos
Santos P. L., Stachelek P., Takeda Y., Pander P. (2024). Recent advances
in highly-efficient near infrared OLED emitters. Mater. Chem. Front..

[16] Bünzli J.-C. G., Eliseeva S. V. (2010). Lanthanide NIR luminescence for telecommunications,
bioanalyses and solar energy conversion. J.
Rare Earths.

[17] Wang H., Gao Y., Chen J., Fan X.-C., Shi Y.-Z., Yu J., Wang K., Li S., Lee C.-S., Zhang X. (2025). Versatile
thermally activated delayed fluorescence material enabling high efficiencies
in both photodynamic therapy and deep-red/NIR electroluminescence. ACS Nano.

[18] Congrave D. G., Drummond B. H., Conaghan P. J., Francis H., Jones S. T. E., Grey C. P., Greenham N. C., Credgington D., Bronstein H. (2019). A simple molecular design strategy for delayed fluorescence
toward 1000 nm. J. Am. Chem. Soc..

[19] Li C., Duan R., Liang B., Han G., Wang S., Ye K., Liu Y., Yi Y., Wang Y. (2017). Deep-red to near-infrared
thermally activated delayed fluorescence in organic solid films and
electroluminescent devices. Angew. Chem., Int.
Ed..

[20] Yuan Y., Hu Y., Zhang Y. X., Lin J. D., Wang Y. K., Jiang Z. Q., Liao L. S., Lee S. T. (2017). Over 10% EQE near-infrared electroluminescence
based on a thermally activated delayed fluorescence emitter. Adv. Funct. Mater..

[21] Wang J., Jiang C., Cao C., Zhuang X., Liang B., Wang Y., Bi H. (2025). Red thermally
activated delayed fluorescence
materials for high-performance organic light-emitting diode. Org. Electron..

[22] Tai J.-W., Tang Y., Zhang K., Yang C.-Z., Pan Z.-H., Lin Y.-C., Shih Y.-W., Chen C.-H., Chiu T.-L., Lee J.-H., Wang C.-K., Wu C.-C., Fan J. (2023). 13.2% EQE
near-infrared TADF OLED with emission peak at 761 nm. Chem. Eng. J..

[23] Cai Z., Wu X., Liu H., Guo J., Yang D., Ma D., Zhao Z., Tang B. Z. (2021). Realizing record-high electroluminescence
efficiency of 31.5% for red thermally activated delayed fluorescence
molecules. Angew. Chem., Int. Ed..

[24] Wang S., Yan X., Cheng Z., Zhang H., Liu Y., Wang Y. (2015). Highly efficient
near-infrared delayed fluorescence organic light emitting diodes using
a phenanthrene-based charge-transfer compound. Angew. Chem., Int. Ed..

[25] Klimash A., Pander P., Klooster W. T., Coles S. J., Data P., Dias F. B., Skabara P. J. (2018). Intermolecular interactions in molecular
crystals and their effect on thermally activated delayed fluorescence
of helicene-based emitters. J. Mater. Chem.
C.

[26] Wang H., Wang K., Chen J. X., Zhang X., Zhou L., Fan X. C., Cheng Y. C., Hao X. Y., Yu J., Zhang X. H. (2023). Enabling record-high
deep-red/near-infrared electroluminescence
through subtly managing intermolecular interactions of a thermally
activated delayed fluorescence emitter. Adv.
Funct. Mater..

[27] Li S., Chen L., Li M., Zhang T., Yan S., Ren Z. (2024). Designing solution-processed
thermally activated delayed fluorescence
emitters via introducing bulky steric hindrance groups for pure red
OLEDs. J. Mater. Chem. C.

[28] Tang Y., He J.-L., Zhang K., Zhao Y., Lin Y.-C., Chen C.-H., Chiu T.-L., Lee J.-H., Wang C.-K., Fan J., Wu C.-C. (2024). Enhancing
emission performance of red TADF emitters
via the introduction of electronically inert pendant. Org. Electron..

[29] Hu S., Li Y., Zhang K., Zhou D.-Y., Liao L.-S., Fan J. (2025). Pentacarbonitrile-based
efficient near-infrared thermally activated delayed fluorescence OLEDs
via suppressing excited-state structural relaxation. J. Mater. Chem. C.

[30] Northey T., Stacey J., Penfold T. J. (2017). The role of solid state solvation
on the charge transfer state of a thermally activated delayed fluorescence
Emitter. J. Mater. Chem. C.

[31] Urban M., Marek-Urban P. H., Durka K., Luliński S., Pander P., Monkman A. P. (2023). TADF invariant
of host polarity and
ultralong fluorescence lifetimes in a donor-acceptor emitter featuring
a hybrid sulfone-triarylboron acceptor. Angew.
Chem., Int. Ed..

[32] Marek-Urban P. H., Natkowski D. R., Wrochna K., Zuba A., Jedrzejczyk G., Blacha-Grzechnik A., Grzywa M., Wozniak K., Durka K. (2024). Luminescence
modulated by molecular conformation in stimuli-responsive polymorphs
of dibenzothiaborinine dioxide-pyridylphenolate complex. Dyes Pigm..

[33] Urban M., Wrochna K., Marek-Urban P. H., Natkowski D. R., Woźniak K., Pander P., Monkman A. P., Durka K., Luliński S. (2025). Strongly fluorescent spiro-type tetracoordinate
complexes
of dibenzo­[*b*,*e*]­[1,4]­thiaborinine
dioxide with functionalized 2-(benzo­[*d*]­heterazol-2-yl)­phenolate
ligands displaying TADF. J. Mater. Chem. C.

[34] Daniels R. E., Culham S., Hunter M., Durrant M. C., Probert M. R., Clegg W., Williams J. A. G., Kozhevnikov V. N. (2016). When two
are better than one: bright phosphorescence from non-stereogenic dinuclear
iridium (III) complexes. Dalton Trans.

[35] Gibson J., Monkman A. P., Penfold T. J. (2016). The importance
of vibronic coupling
for efficient reverse intersystem crossing in thermally activated
delayed fluorescence molecules. ChemPhysChem.

[36] Hall D., Sancho-García J. C., Pershin A., Beljonne D., Zysman-Colman E., Olivier Y. (2023). Benchmarking DFT functionals for
excited-state calculations of donor–acceptor TADF emitters:
insights on the key parameters determining reverse inter-system crossing. J. Phys. Chem. A.

[37] Ivanova G., Bozova N., Petkov N., An C., Hu B., Mutovska M., Konstantinov K., Zagranyarski Y., Videva V., Yordanova A., Baumgarten M., Ivanova A. (2022). Benchmarking of density functionals for the description
of optical properties of newly synthesized π-conjugated TADF
blue emitters. Chem. - Eur. J..

[38] Murawski C., Leo K., Gather M. C. (2013). Efficiency roll-off
in organic light-emitting diodes. Adv. Mater..

[39] Sasaki S., Murakami M., Murakami F., Yoshifuji M. (2011). Synthesis
and redox properties of sterically crowded triarylphosphine and tetraaryldiphosphane
bearing phenothiazinyl groups. Heteroat. Chem..

[40] Pander P., Data P., Turczyn R., Lapkowski M., Swist A., Soloducho J., Monkman A. P. (2016). Synthesis and characterization
of chalcogenophene-based monomers with pyridine acceptor unit. Electrochim. Acta.

[41] Data P., Pander P., Lapkowski M., Swist A., Soloducho J., Reghu R. R., Grazulevicius J. V. (2014). Unusual
properties of electropolymerized
2,7- and 3,6- carbazole derivatives. Electrochim.
Acta.

[42] Cardona C. M., Li W., Kaifer A. E., Stockdale D., Bazan G. C. (2011). Electrochemical
considerations for determining absolute frontier orbital energy levels
of conjugated polymers for solar cell applications. Adv. Mater..

[43] Bredas J.-L. (2014). Mind the
gap!. Mater. Horiz..

[44] Resch-Genger U., Rurack K. (2013). Determination of the
photoluminescence quantum yield
of dilute dye solutions (IUPAC technical report). Pure Appl. Chem..

[45] Würth C., Grabolle M., Pauli J., Spieles M., Resch-Genger U. (2013). Relative and
absolute determination of fluorescence quantum yields of transparent
samples. Nat. Protoc..

[46] Pander P., Data P., Dias F. B. (2018). Time-resolved
photophysical characterization
of triplet-harvesting organic compounds at an oxygen-free environment
using an iCCD camera. J. Visualized Exp..

[47] Pander P., Nastula D., Marek-Urban P. H., Kozhevnikov V. N., Williams J. A. G. (2026). Evidence for thermally activated delayed fluorescence
in iridium­(iii) complexes. Inorg. Chem. Front..

[48] Pereira D. de S., Monkman A. P., Data P. (2018). Production
and characterization of
vacuum deposited organic light emitting diodes. J. Visualized Exp..

[49] Frisch, M. J. ; Trucks, G. W. ; Schlegel, H. B. ; Scuseria, G. E. ; Robb, M. A. ; Cheeseman, J. R. ; Scalmani, G. ; Barone, V. ; Petersson, G. A. ; Nakatsuji, H. ; Li, X. ; Caricato, M. ; Marenich, A. V. ; Bloino, J. ; Janesko, B. G. ; Gomperts, R. ; Mennucci, B. ; Hratchian, H. P. ; Ortiz, J. V. ; Izmaylov, A. F. ; Sonnenberg, J. L. ; Williams-Young, D. ; Ding, F. ; Lipparini, F. ; Egidi, F. ; Goings, J. ; Peng, B. ; Petrone, A. ; Henderson, T. ; Ranasinghe, D. ; Zakrzewski, V. G. ; Gao, J. ; Rega, N. ; Zheng, G. ; Liang, W. ; Hada, M. ; Ehara, M. ; Toyota, K. ; Fukuda, R. ; Hasegawa, J. ; Ishida, M. ; Nakajima, T. ; Honda, Y. ; Kitao, O. ; Nakai, H. ; Vreven, T. ; Throssell, K. ; Montgomery, J. A., Jr. ; Peralta, J. E. ; Ogliaro, F. ; Bearpark, M. J. ; Heyd, J. J. ; Brothers, E. N. ; Kudin, K. N. ; Staroverov, V. N. ; Keith, T. A. ; Kobayashi, R. ; Normand, J. ; Raghavachari, K. ; Rendell, A. P. ; Burant, J. C. ; Iyengar, S. S. ; Tomasi, J. ; Cossi, M. ; Millam, J. M. ; Klene, M. ; C. Adamo, C. ; Cammi, R. ; Ochterski, J. W. ; Martin, R. L. ; Morokuma, K. ; Farkas, O. ; Foresman, J. B. ; Fox, D. J. ; Gaussian 16, Gaussian, Inc: Wallingford, CT, 2016.

[50] Vosko S. H., Wilk L., Nusair M. (1980). Accurate spin-dependent electron
liquid correlation energies for local spin density calculations: a
critical analysis. Can. J. Phys..

[51] Becke A. D. (1993). Density-functional
thermochemistry. III. The role of exact exchange. J. Chem. Phys..

[52] Lee C., Yang W., Parr R. G. (1988). Development of the Colle-Salvetti
correlation-energy formula into a functional of the electron density. Phys. Rev. B.

[53] Krishnan R., Binkley J. S., Seeger R., Pople J. A. (1980). Self-consistent
mlecular orbital methods. XX. A basis set for correlated wave functions. J. Chem. Phys..

[54] Yanai T., Tew D. P., Handy N. C. (2004). A new hybrid exchange–correlation
functional using the coulomb-attenuating method (CAM-B3LYP). Chem. Phys. Lett..

[55] Martin R. L. (2003). Natural
transition orbitals. J. Chem. Phys..

[56] Hanwell M. D., Curtis D. E., Lonie D. C., Vandermeersch T., Zurek E., Hutchison G. R. (2012). Avogadro:
an advanced semantic chemical
editor, visualization, and analysis platform. J. Cheminf..

[57] Neese F. (2012). The ORCA program
system. Wiley Interdiscip. Rev.: Comput. Mol.
Sci..

