# Supplementary material for: Enhancing the Red and Near Infrared OLED Efficiency of a TADF Emitter through an Internal Solvation Effect
Source: ACS Appl Mater Interfaces. 2026 Apr 29;18(18):26569–78. doi: 10.1021/acsami.6c01458 (PMC13298805; doi:10.1021/acsami.6c01458)
Supplement: Supplementary file 1 [file am6c01458_si_001.pdf]

Supporting Information

Enhancing Red and Near Infrared OLED Efficiency of a TADF Emitter Through Internal Solvation Effect

Wojciech Derkowski,[a] Piotr Pander,[b,c*] Adam Zuba,[a] Krzysztof Durka,[a*] Sergiusz Luliński[a*] and Andrew P. Monkman [d]

[a] Faculty of Chemistry, Warsaw University of Technology, Noakowskiego 3, 00-664 Warsaw, Poland
[b] Faculty of Chemistry, Silesian University of Technology, Strzody 9, 44-100 Gliwice, Poland
[c] Centre for Organic and Nanohybrid Electronics, Silesian University of Technology, Konarskiego 22B, 44-100 Gliwice, Poland
[d] Department of Physics, Durham University, South Road, Durham DH1 3LE, UK

Please direct e-mail correspondence to:
Piotr Pander: piotr.pander@polsl.pl
Krzysztof Durka: krzysztof.durka@pw.edu.pl
Sergiusz Luliński: sergiusz.lulinski@pw.edu.pl

Table of contents

1. Crystal structures 2
2. Photophysics 6
3. Electrochemical data 14
4. Thermal characterisation 15
5. Quantum chemical calculations 16
6. Electroluminescent devices 19
7. NMR and HRMS spectra 22

1. Crystal structures

Table S1. Selected crystal data, data collection and refinement parameters for **PTZ-Dipp-SO2B** and **PTZ-Dipp-(Bu)SO2B**.

	**PTZ-Dipp-SO2B**	**PTZ-Dipp-(Bu)SO2B**
Empirical formula	$C_{37}H_{34}NO_2S_2BCl_2$	$C_{40}H_{40}NO_2S_2B$
Formula weight	670.48	641.66
T / K	100.0(1)	100.0(1)
Crystal system	monoclinic	orthorhombic
Space group	$P2_1$	$P2_12_12_1$
a / Å	9.65770(10)	10.7820(4)
b / Å	8.39060(10)	14.1265(6)
c / Å	21.0566(3)	22.8337(7)
α / °	90	90
β / °	100.5010(10)	90
γ / °	90	90
Volume / Å^3	1677.72(4)	3477.8(2)
Z	2	4
ρ_{calc} / g·cm^{-3}	1.327	1.225
μ / mm^{-1}	3.170	0.189
F(000)	700.0	1360.0
Crystal size / mm^3	0.422 × 0.165 × 0.055	0.246 × 0.185 × 0.148
Radiation	Cu Kα (λ = 1.54184)	Mo Kα (λ = 0.71073)
2Θ /°	9.312 to 155.534	4.178 to 58.906
Index ranges	$-12 \leq h \leq 12$, $-10 \leq k \leq 10$, $-26 \leq l \leq 26$	$-14 \leq h \leq 14$, $-18 \leq k \leq 18$, $-31 \leq l \leq 30$
Reflections collected	20971	24025
Independent reflections	6226 [R_{int} = 0.0288, R_{sigma} = 0.0257]	24025 [R_{int} = 0.0317, R_{sigma} = 0.0592]
Data/restraints/parameters	6226/1/406	24025/0/417
Goodness-of-fit on F^2	1.091	1.107
Final R indexes [I>=2σ (I)]	R_1 = 0.0824, wR_2 = 0.2260	R_1 = 0.0690, wR_2 = 0.1455
Final R indexes [all data]	R_1 = 0.0836, wR_2 = 0.2277	R_1 = 0.0779, wR_2 = 0.1505
Largest diff. peak/hole / e·Å^{-3}	1.46 / −0.81	0.78 / −0.56

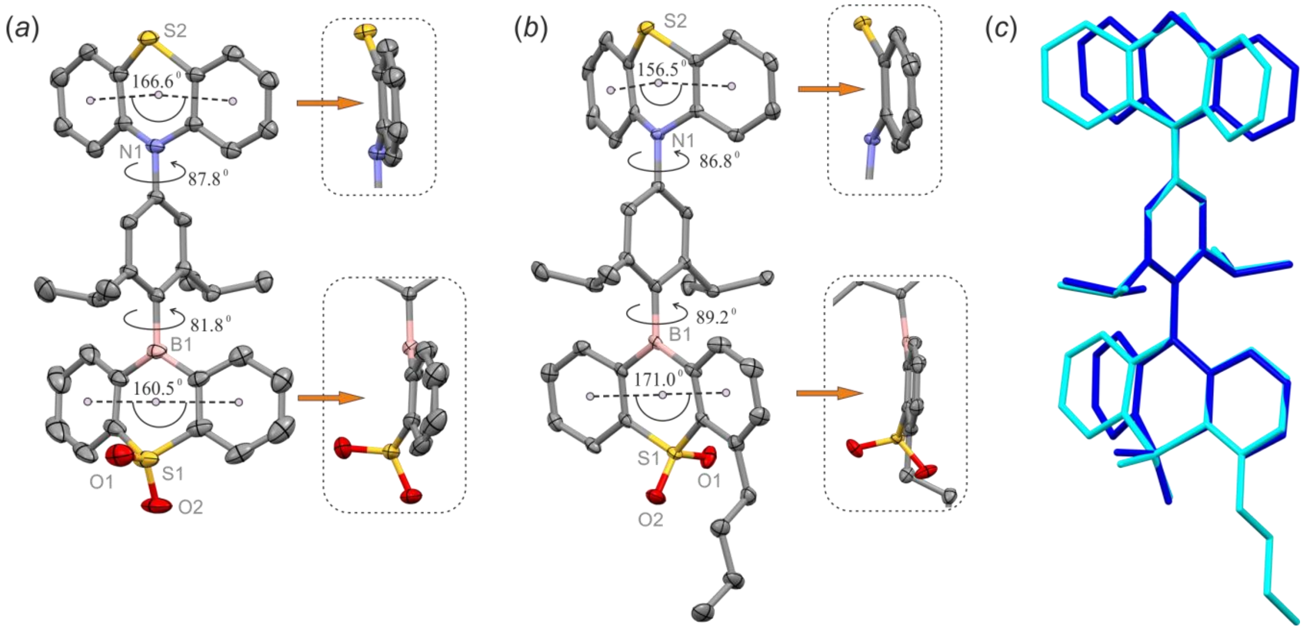

Figure S1. Molecular structures of (*a*) **PTZ-Dipp-SO2B** and (*b*) **PTZ-Dipp-(Bu)SO2B** determined by single-crystal X-ray diffraction. Ellipsoids drawn at the 50% probability level. Hydrogen atoms and DCM solvent molecule (**PTZ-Dipp-SO2B**) are omitted for clarity. (c) Overlay of molecular geometries of **PTZ-Dipp-SO2B** (blue) and **PTZ-Dipp-(Bu)SO2B** (cyan).

Table S2. Basic geometrical parameters in **PTZ-Dipp-SO2B** and **PTZ-Dipp-(Bu)SO2B**. *d* refers to bond distances, *α* bond angles and *τ* torsion angles; parameters are defined on given scheme.

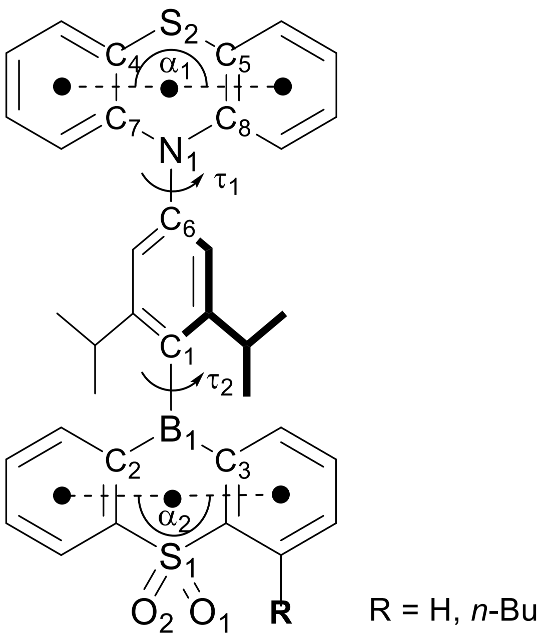

	PTZ-Dipp-SO2B	PTZ-Dipp-(Bu)SO2B
d_{B1-C1} / Å	1.581(7)	1.574(7)
d_{B1-C2} / Å	1.564(9)	1.549(8)
d_{B1-C3} / Å	1.562(8)	1.566(8)
d_{S1-O1} / Å	1.440(6)	1.443(4)
d_{S1-O2} / Å	1.428(7)	1.435(4)
d_{S2-C4} / Å	1.772(5)	1.754(5)
d_{S2-C5} / Å	1.767(5)	1.770(5)
d_{N1-C6} / Å	1.448(6)	1.450(6)
d_{N1-C7} / Å	1.421(6)	1.425(6)
d_{N1-C8} / Å	1.411(6)	1.417(6)
α_1 / °	166.6(5)	156.5(6)
α_2 / °	160.5(5)	171.0(6)
τ_1 / °	87.8(5)	86.8(5)
τ_2 / °	81.8(5)	89.2(5)

Table S3. Geometry of intermolecular interactions in crystal structures of **PTZ-Dipp-SO2B** and **PTZ-Dipp-(Bu)SO2B**.

	Interaction	$d_{C...X}$ / Å	$d_{H...X}$ / Å	$\alpha_{C\text{-}H...X}$ / Å	Symmetry(#)
**PTZ-Dipp-SO2B**	C35-H35…C32#	3.723(7)	2.775	152.5	-x,1/2+y,1-z
	C35-H35…C33#	3.650(9)	2.781	176.2	-x,1/2+y,1-z
	C20-H20C…C25#	3.573(8)	2.744	142.6	x,-1+y,z
	C21-H21B…C31#	3.702(8)	2.870	143.3	x,-1+y,z
	C24-H24B…C2#	3.44(1)	2.962	110.3	x,1+y,z
	C24-H24B…C3#	3.42(1)	2.953	111.3	x,1+y,z
	C9-H9…O2#	3.22(1)	2.427	141.3	1-x,-1/2+y,2-z
	C3-H3…Cl1#	3.718(9)	2.910	143.6	x,-1+y,z
	C37-H37A…O1#	3.15(1)	2.425	130.0	2-x,1/2+y,2-z
	O1…Cl1#	3.109(7)	-	-	x,-1+y,z
**PTZ-Dipp-(Bu)SO2B**	C20-H20B…C27#	3.908(8)	2.965	161.9	1/2+x,1/2-y,1-z
	C21-H21B…C31#	3.706(7)	2.897	140.4	1/2+x,1/2-y,1-z
	C20-H20A…S2#	3.591(7)	2.673	156.1	1+x,y,z
	C21-H21C…S2#	3.708(5)	2.843	147.7	1+x,y,z
	C22-H22B…O1#	3.598(8)	2.669	158.3	-1+x,y,z
	C27-H27…C33#	3.688(7)	2.808	137.6	-1/2+x,1/2-y,1-z
	C35-H35…O2#	3.240(7)	2.678	118.4	1.5-x,1-y,1/2+z
	C40-H40B…C34#	3.813(8)	2.915	152.9	1.5-x,1-y,-1/2+z
	C30…C9#	3.472(7)	-	-	1-x,-1/2+y,1/2-z
	C28…C11#	3.446(7)	-	-	1-x,-1/2+y,1/2-z

2. Photophysics

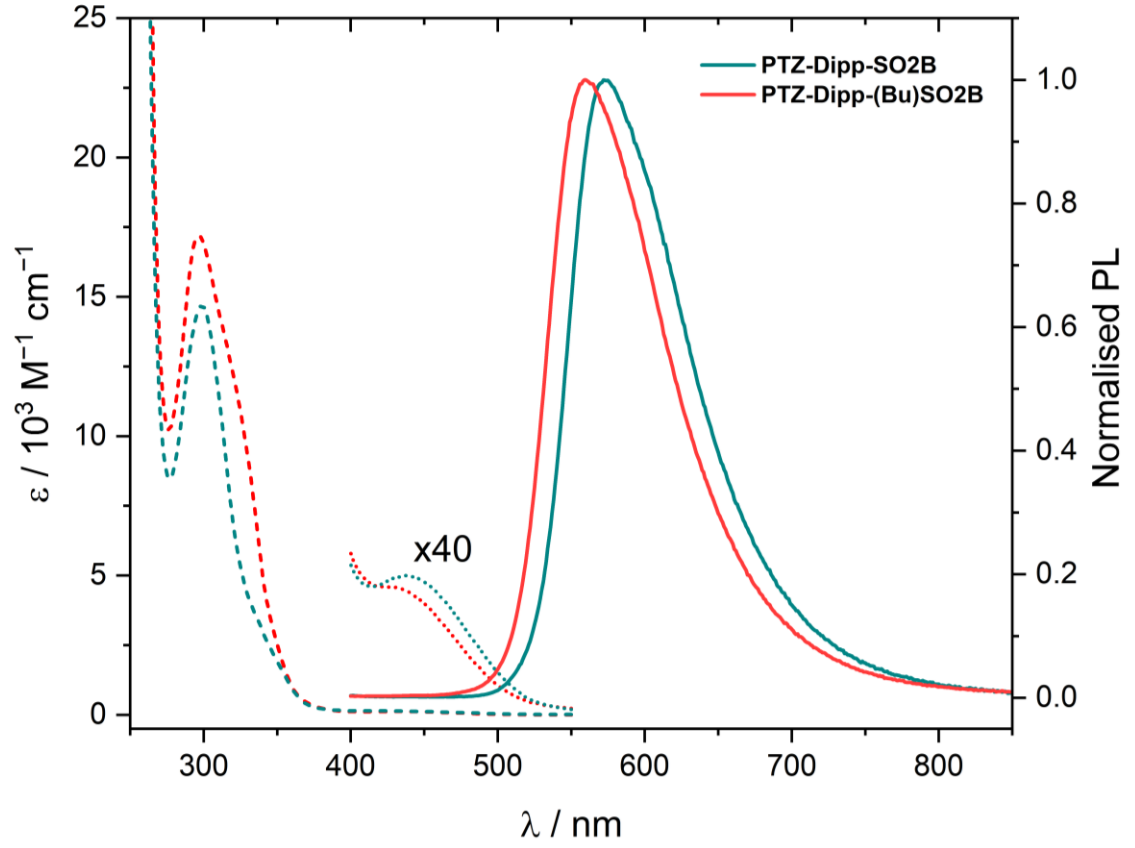

Figure S2. Steady state absorption and photoluminescence (λ_{exc} = 300 nm) spectra of **PTZ-Dipp-SO2B** and **PTZ-Dipp-(Bu)SO2B** in dilute methylcyclohexane solution.

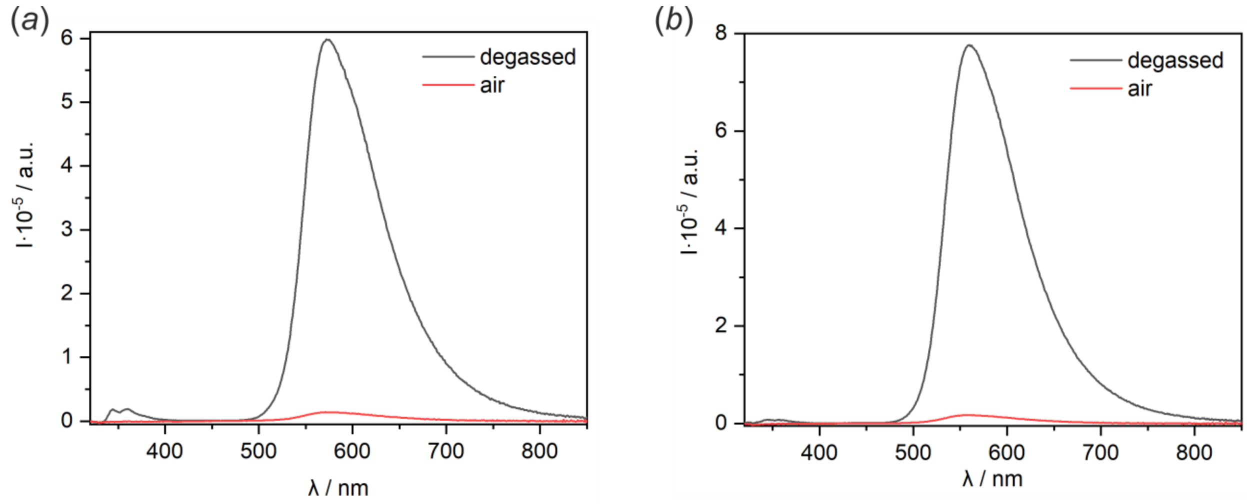

Figure S3. Emission spectra of (*a*) **PTZ-Dipp-SO2B** and (*b*) **PTZ-Dipp-(Bu)SO2B** under air-equilibrated conditions and upon degassing in methylcyclohexane (λ_{ex} = 300 nm).

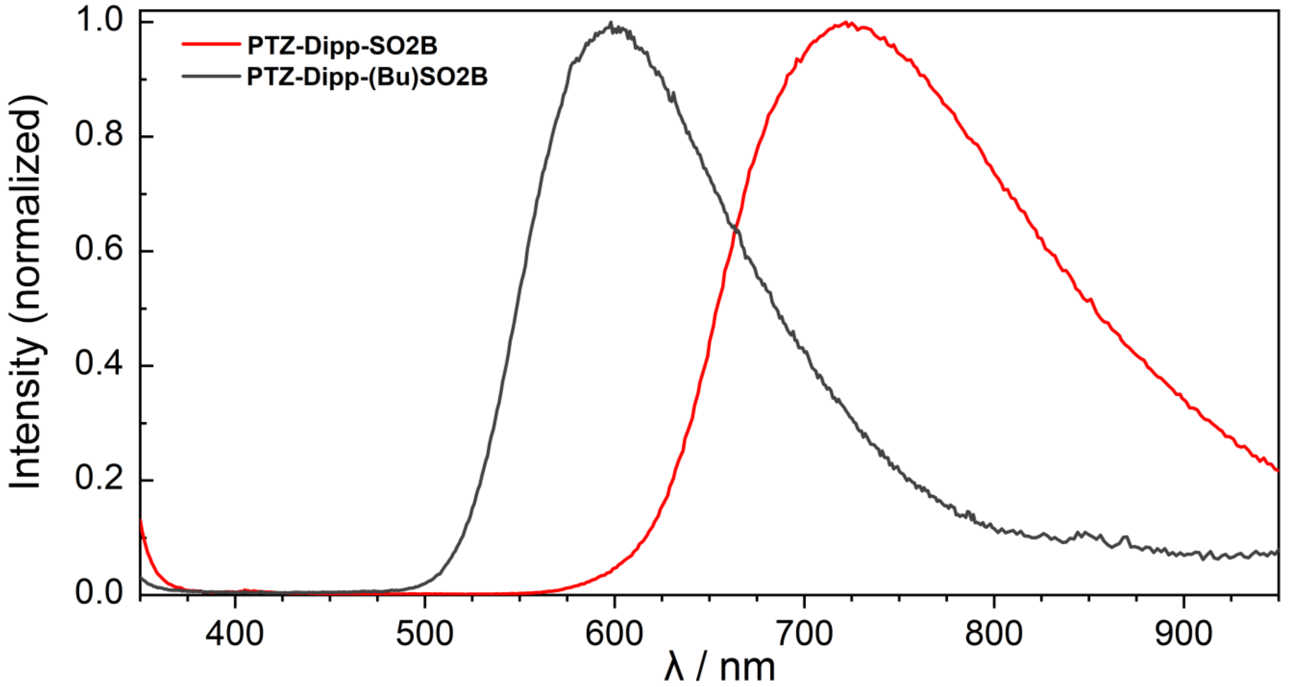

Figure S4. Steady state photoluminescence spectra of **PTZ-Dipp-SO2B** and **PTZ-Dipp-(Bu)SO2B** in solid state (λ_{ex} = 340 nm).

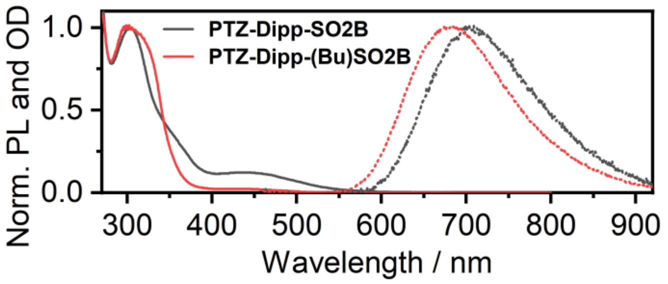

Figure S5. Steady-state absorption and photoluminescence spectra of **PTZ-Dipp-SO2B** and **PTZ-Dipp-(Bu)SO2B** in neat films.

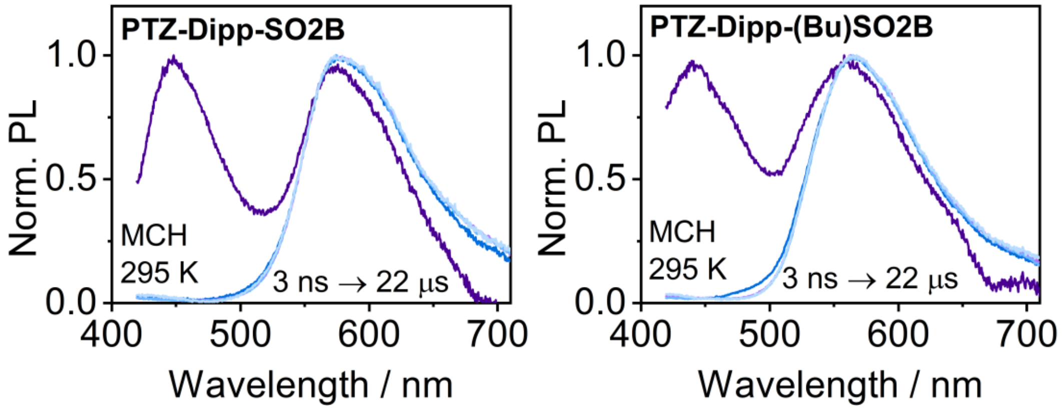

Figure S6. Time-resolved photoluminescence spectra of **PTZ-Dipp-SO2B** and **PTZ-Dipp-(Bu)SO2B** in methylcyclohexane (MCH) at 295 K, c = 10^{-5} M.

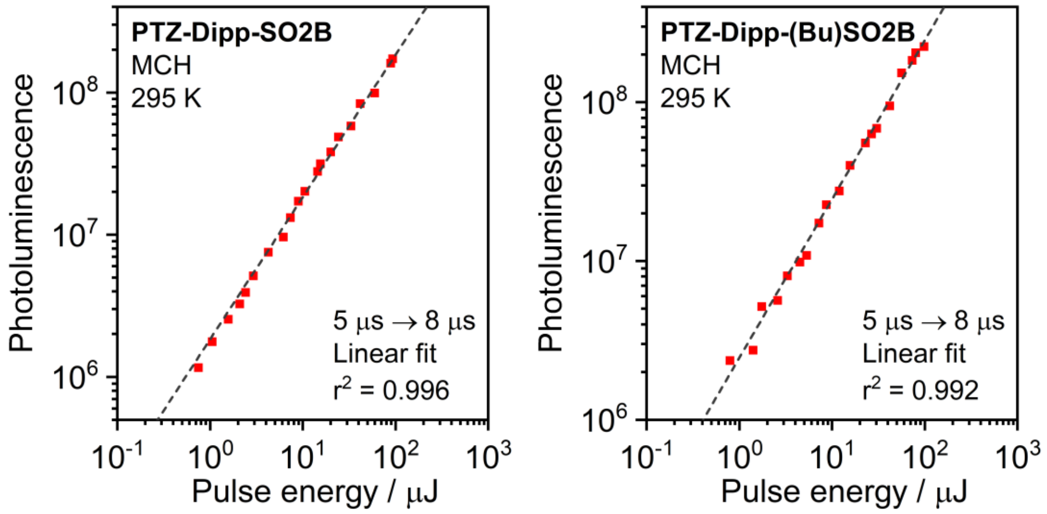

Figure S7. Relationship between the intensity of delayed fluorescence of **PTZ-Dipp-SO2B** and **PTZ-Dipp-(Bu)SO2B** in methylcyclohexane (c = 10^{-5} M), and excitation dose, presented in a double logarithmic scale. Numerical values in the bottom right corner of each individual figure identify the delay time window used in the experiment and the correlation factor r.

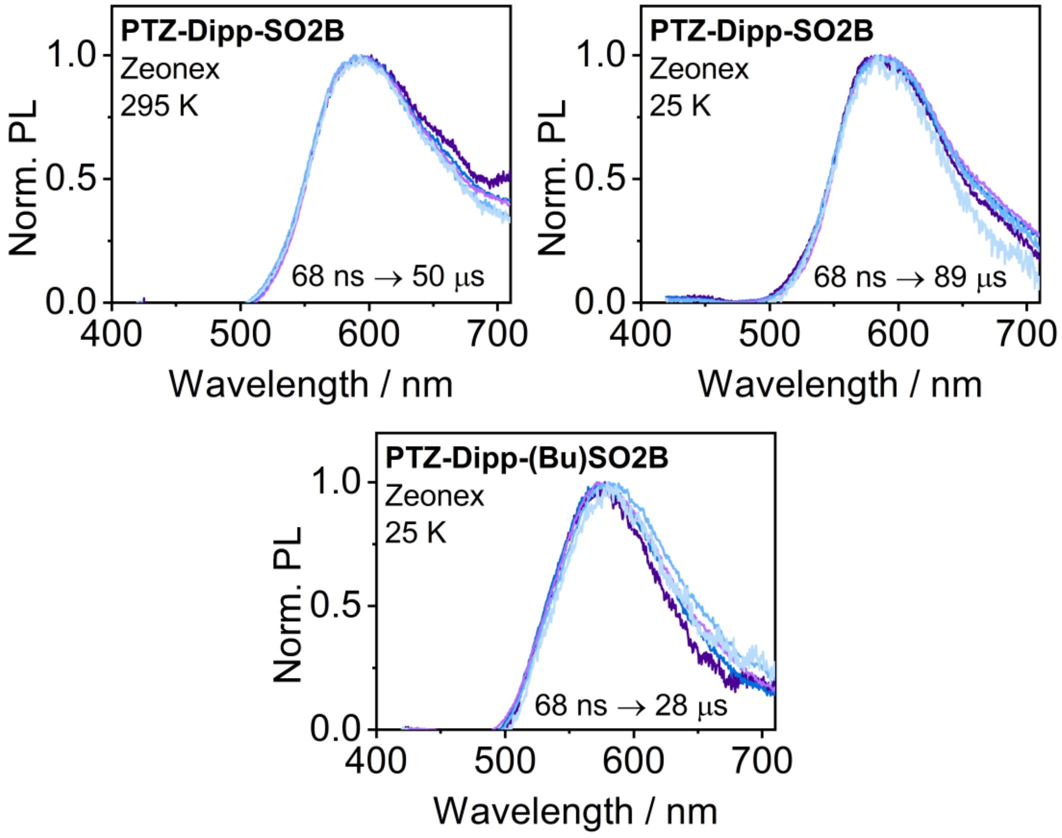

Figure S8. Time-resolved photoluminescence spectra of **PTZ-Dipp-SO2B** and **PTZ-Dipp-(Bu)SO2B** in zeonex (1% w/w) at 295 and 25 K. Note that the set of spectra for **PTZ-Dipp-(Bu)SO2B** at 295 K is shown in the main text, **Figure 2b**.

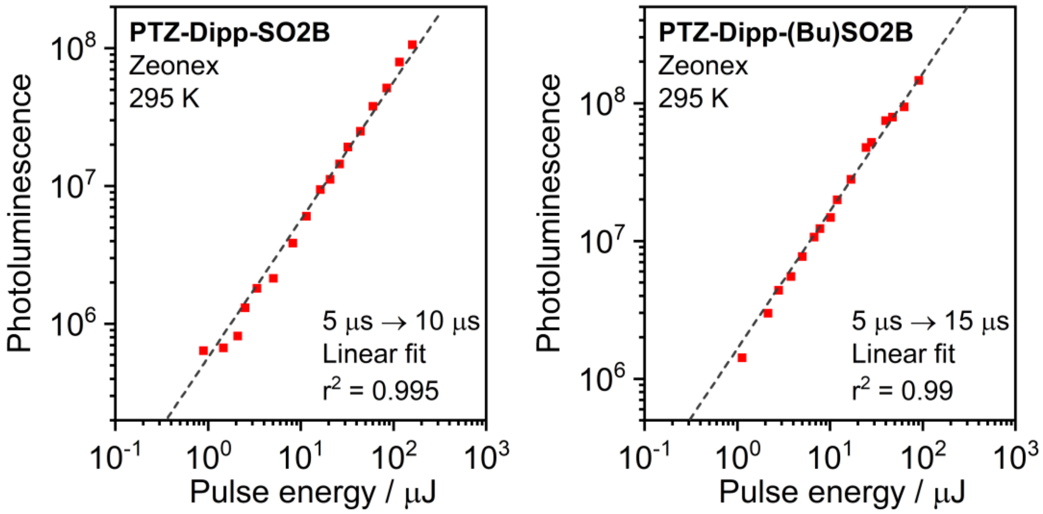

Figure S9. Relationship between the intensity of delayed fluorescence of **PTZ-Dipp-SO2B** and **PTZ-Dipp-(Bu)SO2B** in 1% (w/w) film in zeonex, and excitation dose, presented in a double logarithmic scale. Numerical values in the bottom right corner of each individual figure identify the delay time window used in the experiment and the correlation factor r.

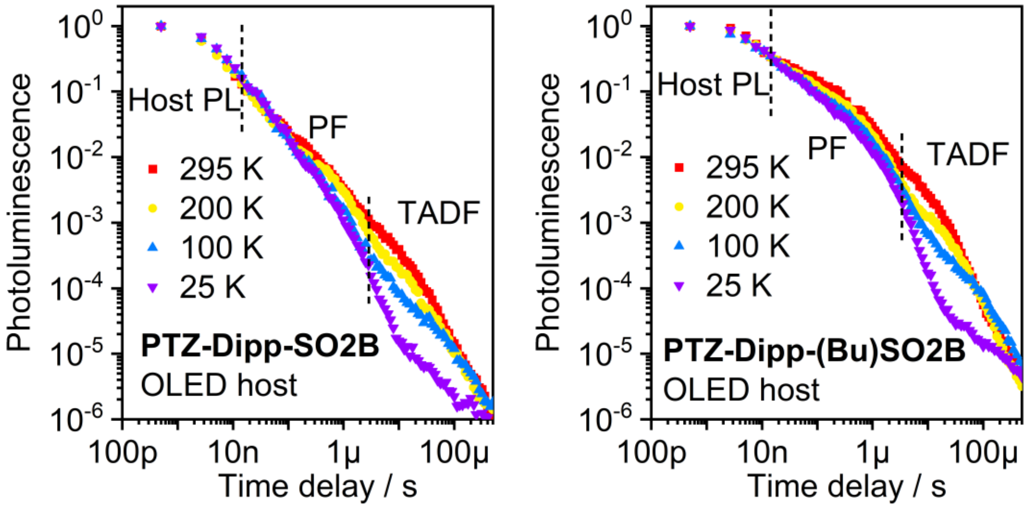

Figure S10. Photoluminescence decay traces in films of **PTZ-Dipp-SO2B** and **PTZ-Dipp-(Bu)SO2B** in OLED host at 7% w/w loading, recorded at temperatures indicated in each figure legend.

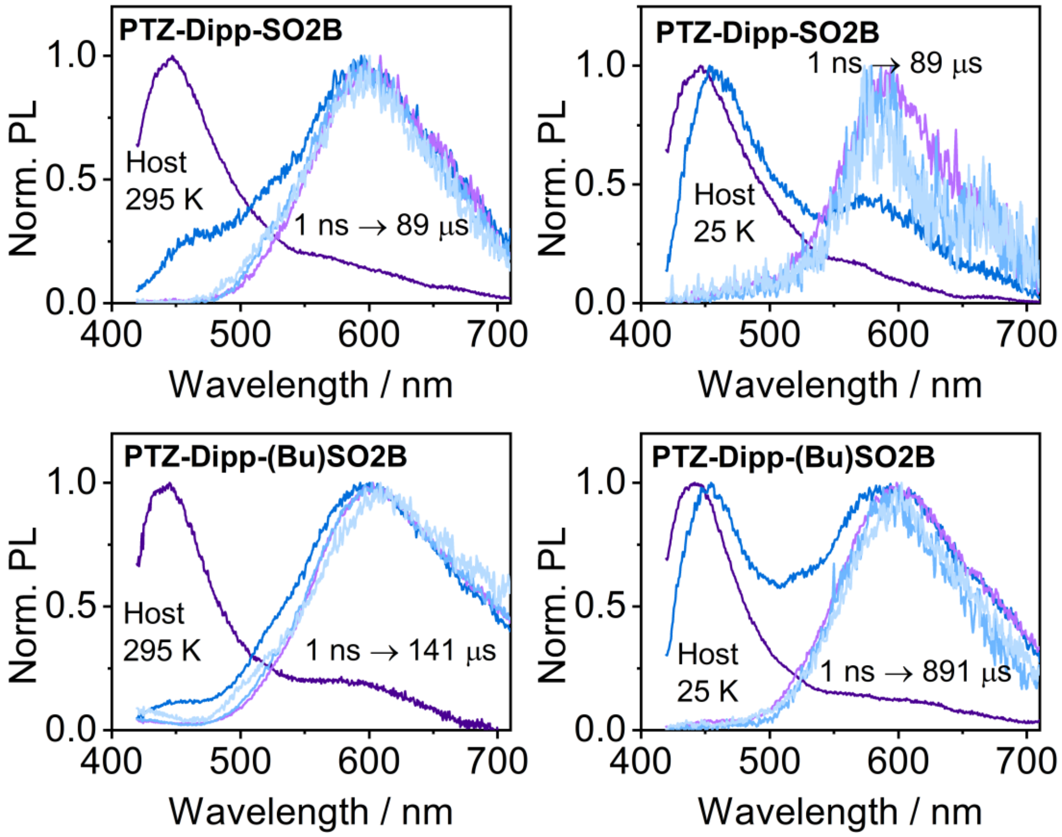

Figure S11. Time-resolved photoluminescence spectra of **PTZ-Dipp-SO2B** and **PTZ-Dipp-(Bu)SO2B** in OLED host at 7% w/w loading at 295 and 25 K.

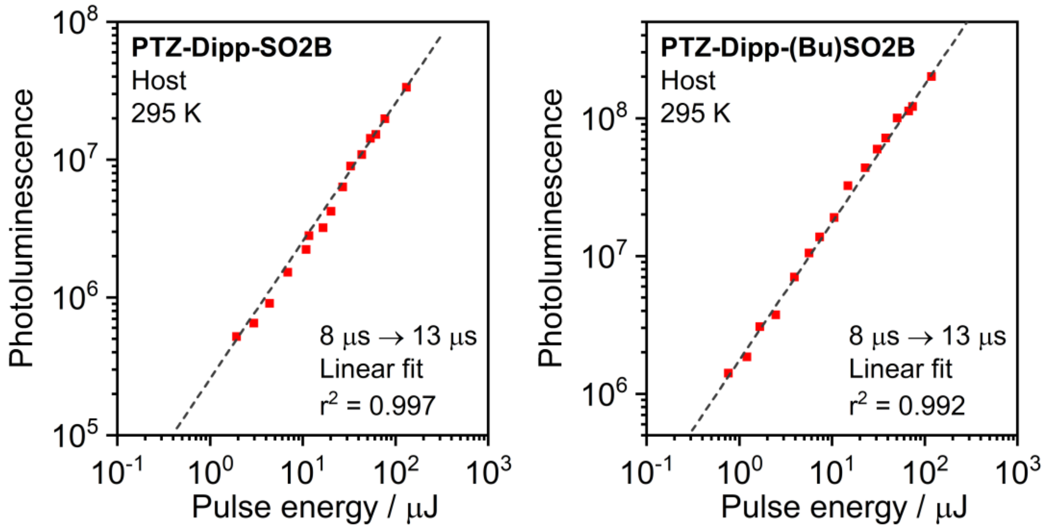

Figure S12. Relationship between the intensity of delayed fluorescence in film of **PTZ-Dipp-SO2B** and **PTZ-Dipp-(Bu)SO2B** at 7% (w/w) loading in OLED host, and excitation dose, presented in a double logarithmic scale. Numerical values in the bottom right corner of each individual figure identify the delay time window used in the experiment and the correlation factor r.

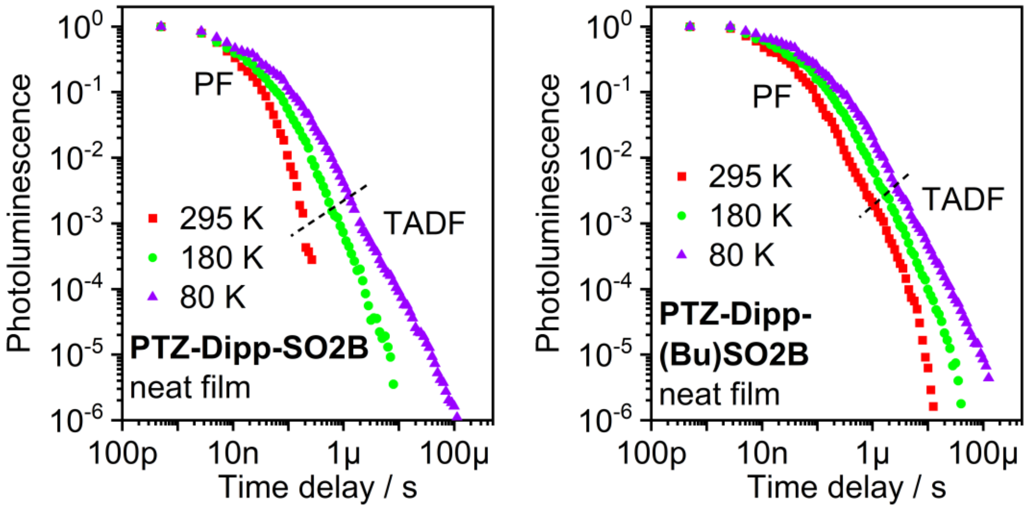

Figure S13. Photoluminescence decay traces in neat films of **PTZ-Dipp-SO2B** and **PTZ-Dipp-(Bu)SO2B**, recorded at temperatures indicated in each figure legend.

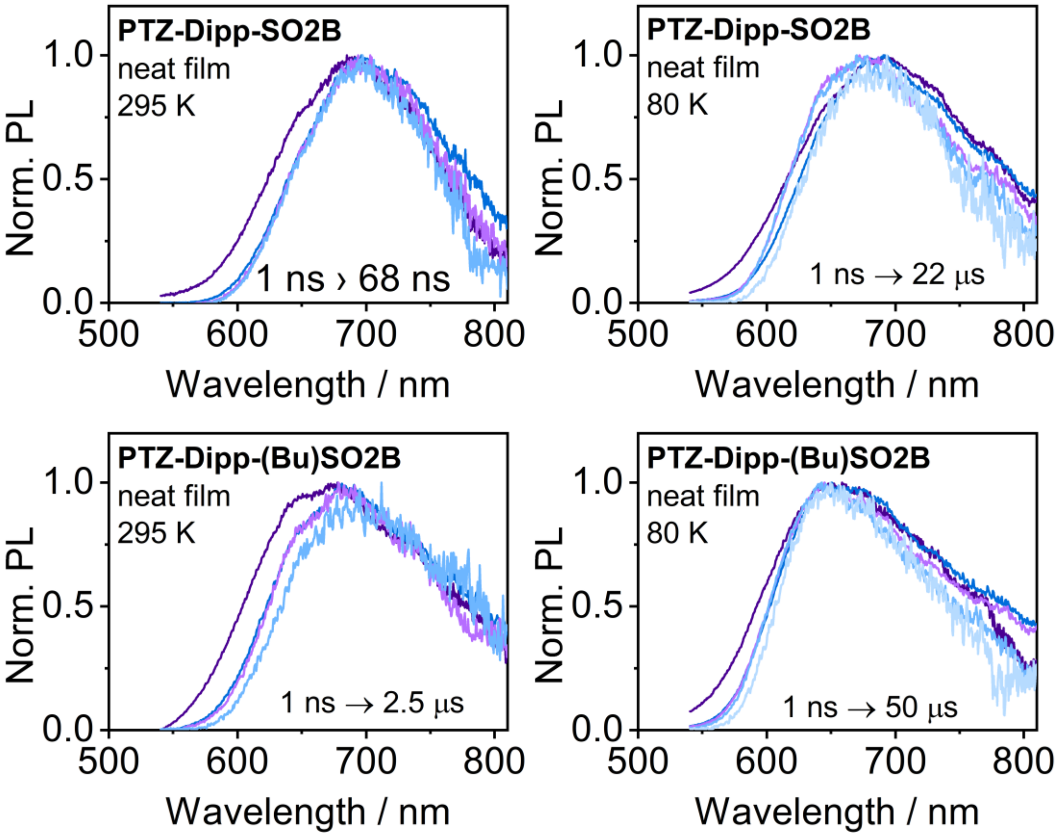

Figure S14. Time-resolved photoluminescence spectra of **PTZ-Dipp-SO2B** and **PTZ-Dipp-(Bu)SO2B** in neat film at 295 and 80 K.

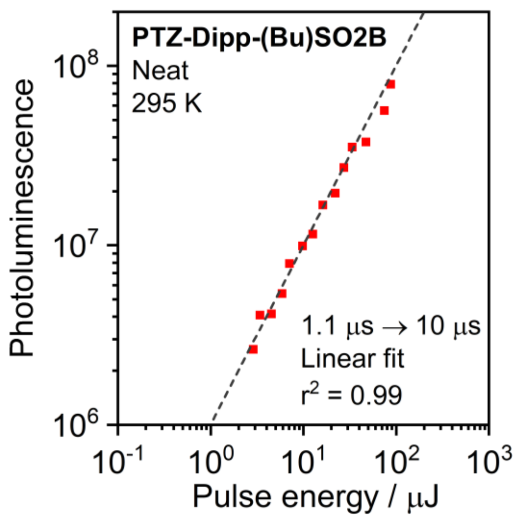

Figure S15. Relationship between the intensity of delayed fluorescence in neat film of **PTZ-Dipp-(Bu)SO2B** and excitation dose, presented in a double logarithmic scale. Numerical values in the bottom right corner identify the delay time window used in the experiment and the correlation factor r.

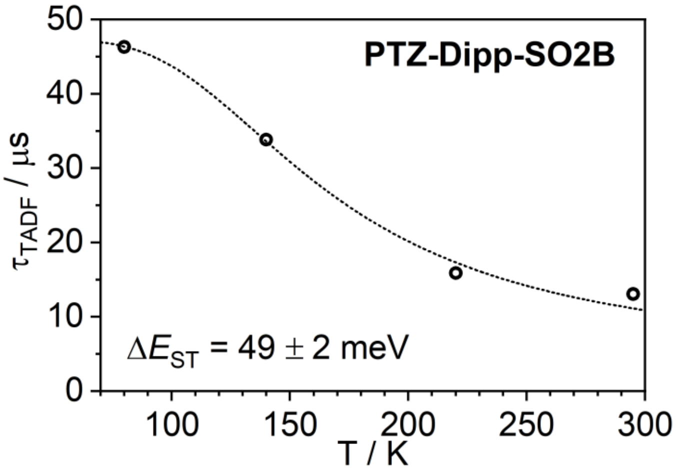

Figure S16. TADF lifetime in Zeonex as a function of temperature in **PTZ-Dipp-SO2B** and the best fit according to equation S1. Note that τ_S is assumed from the decay at 295 K, hence reducing the number of variables to 2.

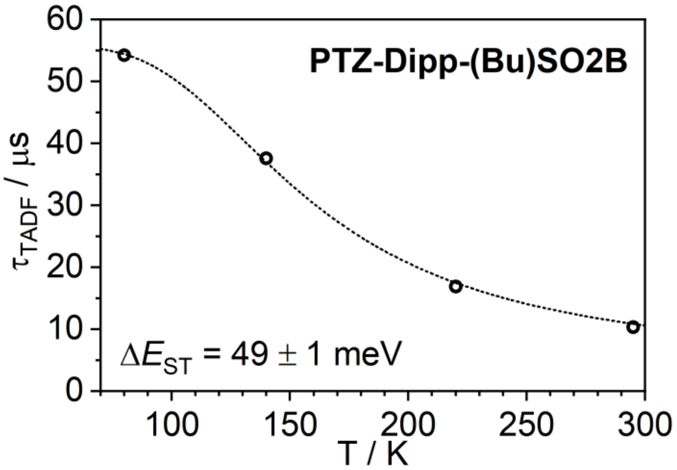

Figure S17. TADF lifetime in Zeonex as a function of temperature in **PTZ-Dipp-(Bu)SO2B** and the best fit according to equation S1. Note that τ_S is assumed from the decay at 295 K, hence reducing the number of variables to 2.

$$\tau_{TADF}(T) = \frac{3 + e^{-\frac{\Delta E_{ST}}{RT}}}{\frac{1}{\tau_T} + \frac{1}{\tau_S} e^{-\frac{\Delta E_{ST}}{RT}}} \qquad \textbf{(S1)}$$

Where $\tau_{TADF}(T)$ – TADF lifetime as a function of temperature T, s; T – temperature, K; ΔE_{ST} – S_1-T_1 energy gap, eV; τ_T – phosphorescence lifetime, s; τ_S – fluorescence lifetime, s.

3. Electrochemical data

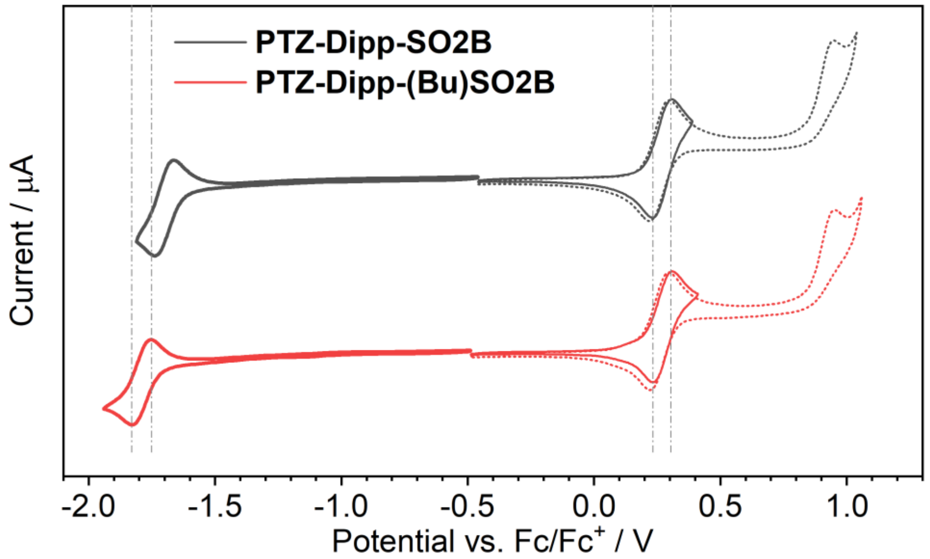

Figure S18. Electrochemical redox processes recorded for **PTZ-Dipp-SO2B** and **PTZ-Dipp-(Bu)SO2B** with cyclic voltammetry at a scan rate of 50 mV s^{-1}. All potentials are referenced to the half-wave potential of the Fc/Fc^+ redox pair.

Table S4. Summary of electrochemical redox potentials recorded for **PTZ-Dipp-SO2B** and **PTZ-Dipp-(Bu)SO2B** with cyclic voltammetry at a scan rate of 50 mV s^{-1}. All potentials are referenced to the half-wave potential of the Fc/Fc^+ redox pair.

**Compound**	**$E_{1/2}^{ox1}$, V[a]**	**$E_{1/2}^{ox2}$, V[a]**	**$E_{1/2}^{red}$, V[b]**	**E_{onset}^{ox1}, V[c]**	**E_{onset}^{ox2}, V[c]**	**E_{onset}^{red}, V[d]**	**IP, eV[e]**	**EA, eV[f]**
**PTZ-Dipp-SO2B**	0.27	0.89	-1.70	0.20	0.85	-1.62	5.30	3.48
**PTZ-Dipp-(Bu)SO2B**	0.27	0.89	-1.79	0.20	0.85	-1.72	5.30	3.38

[a] Oxidation half-wave potential, V; [b] Reduction half-wave potential, V; [c] Oxidation onset potential, V; [d] Reduction onset potential, V; [e] Ionization potential, IP = $e[E_{onset}^{ox}] + 5.1$, eV; [f] Electron affinity, EA = $e[E_{onset}^{red}] + 5.1$, eV.

4. Thermal characterisation

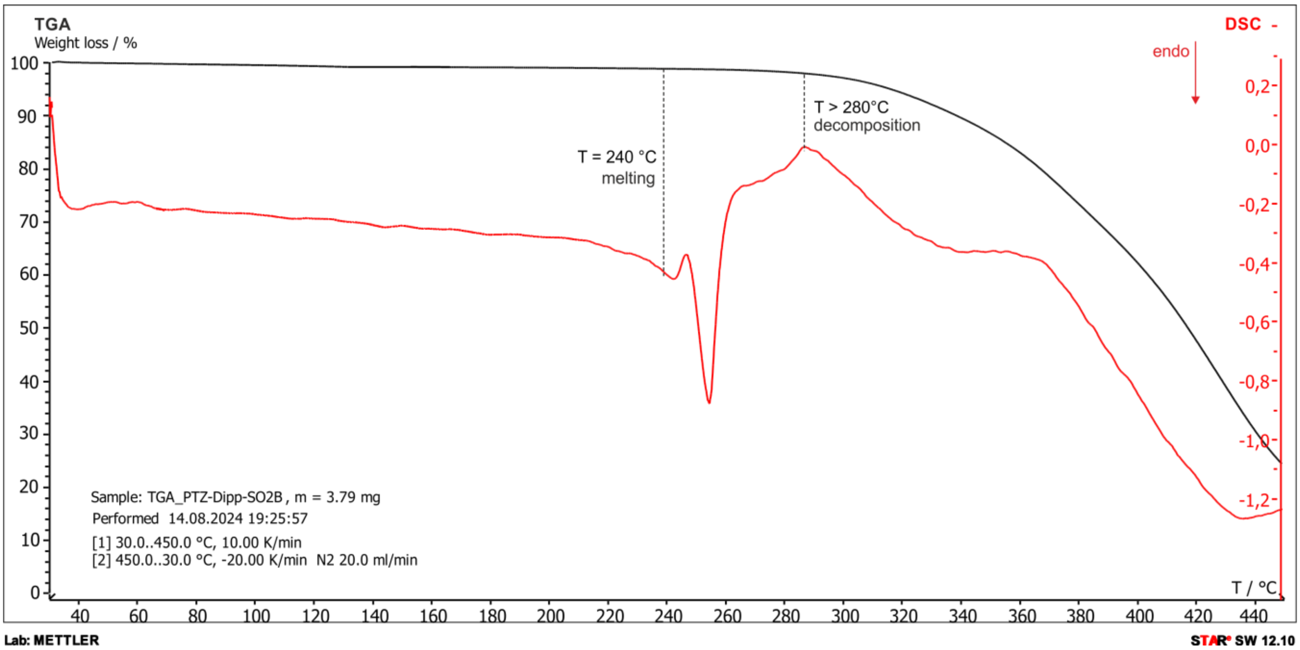

Figure S19. TGA curve for **PTZ-Dipp-SO2B** recorded with the heating rate of 10 K/min.

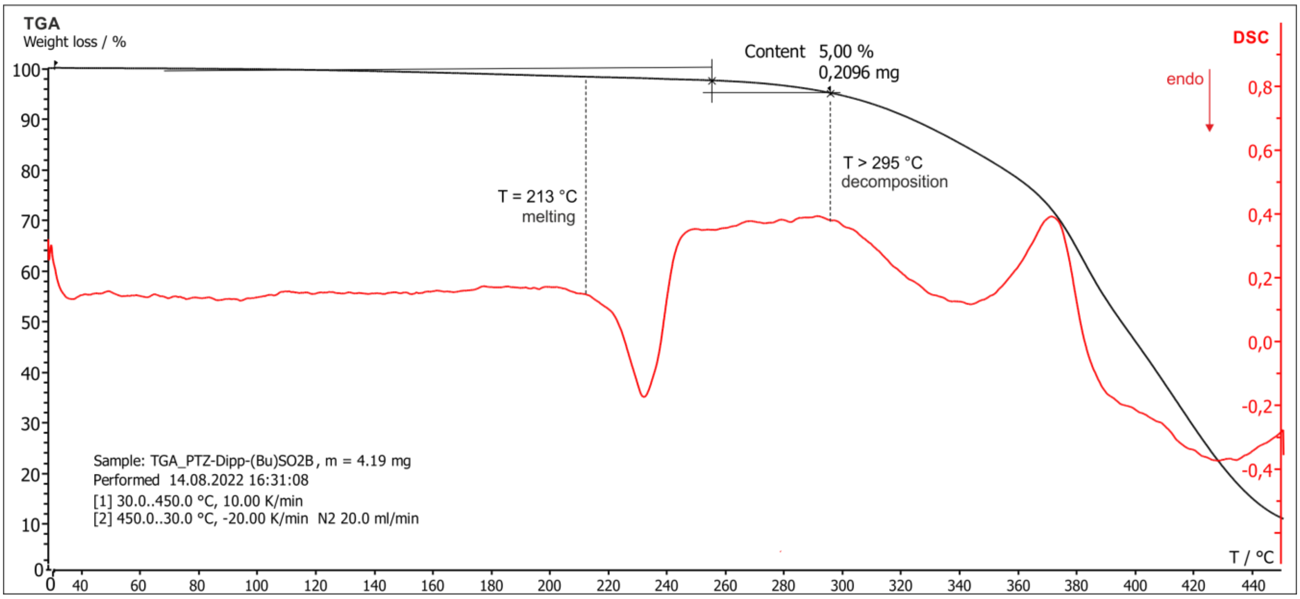

Figure S20. TGA curve for **PTZ-Dipp-(Bu)SO2B** recorded with the heating rate of 10 K/min.

5. Quantum chemical calculations

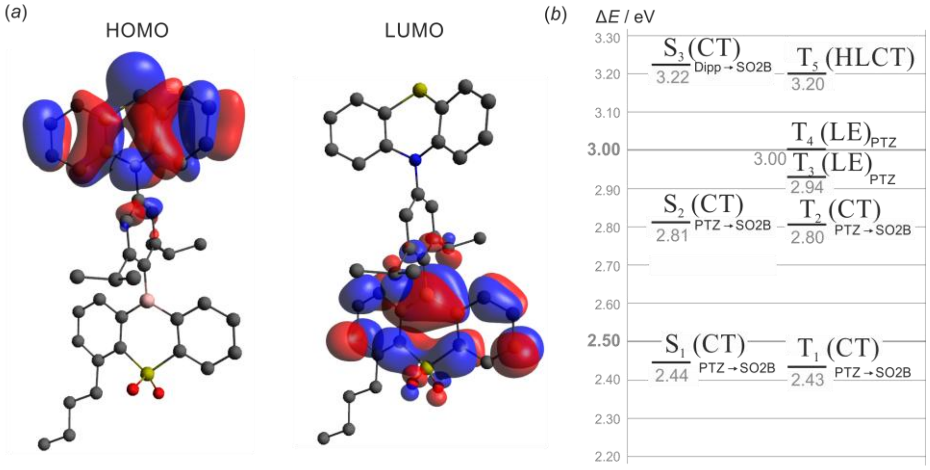

Figure S21. (*a*) Frontier molecular orbital contours (0.02 a.u.) and (*b*) excited state energy diagrams for **PTZ-Dipp-(Bu)SO2B**.

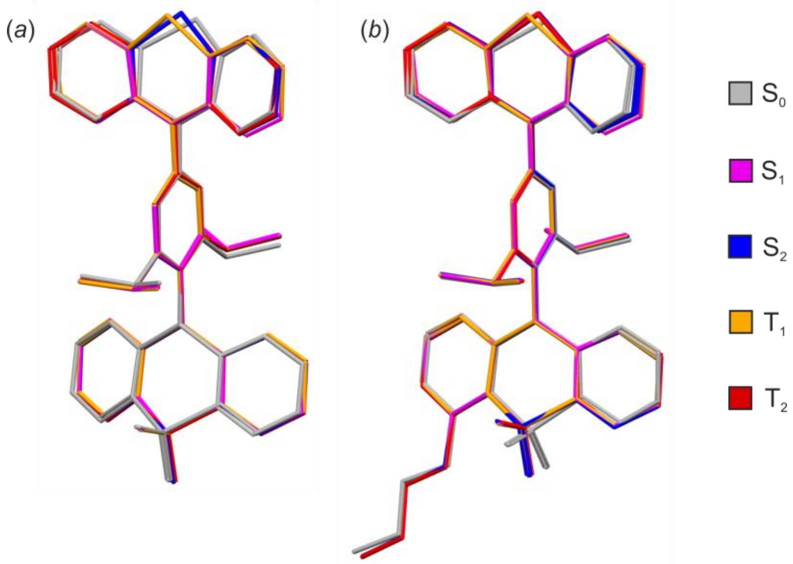

Figure S22. Overlay of molecular structures of (*a*) **PTZ-Dipp-SO2B** and (*b*) **PTZ-Dipp-(Bu)SO2B** after optimisations *in vacuo*: in ground state (gray) and S1 (magenta), S2 (blue), T1 (orange), T2 (red) excited states.

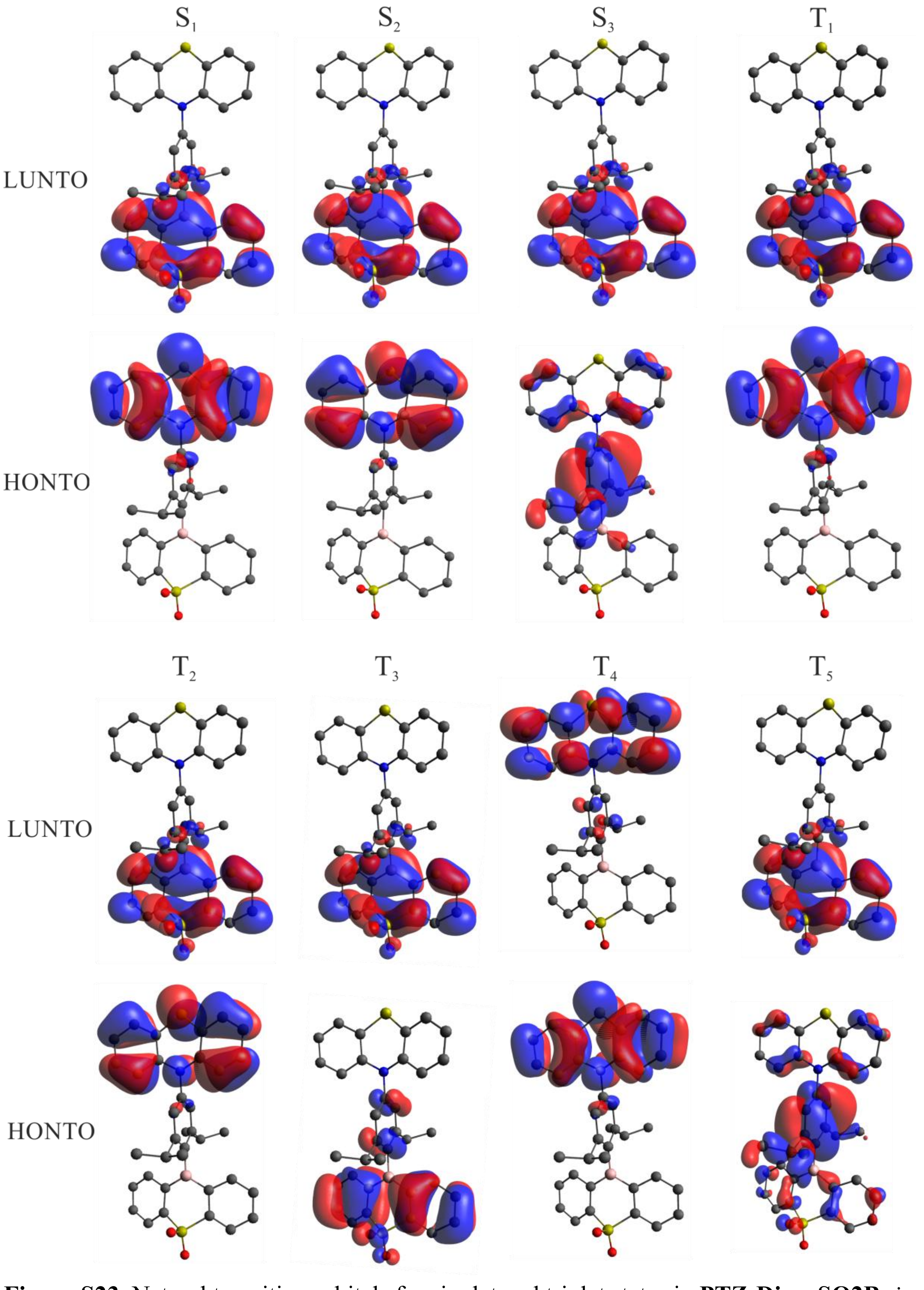

Figure S23. Natural transition orbitals for singlet and triplet states in **PTZ-Dipp-SO2B**; *iso* = 0.02.

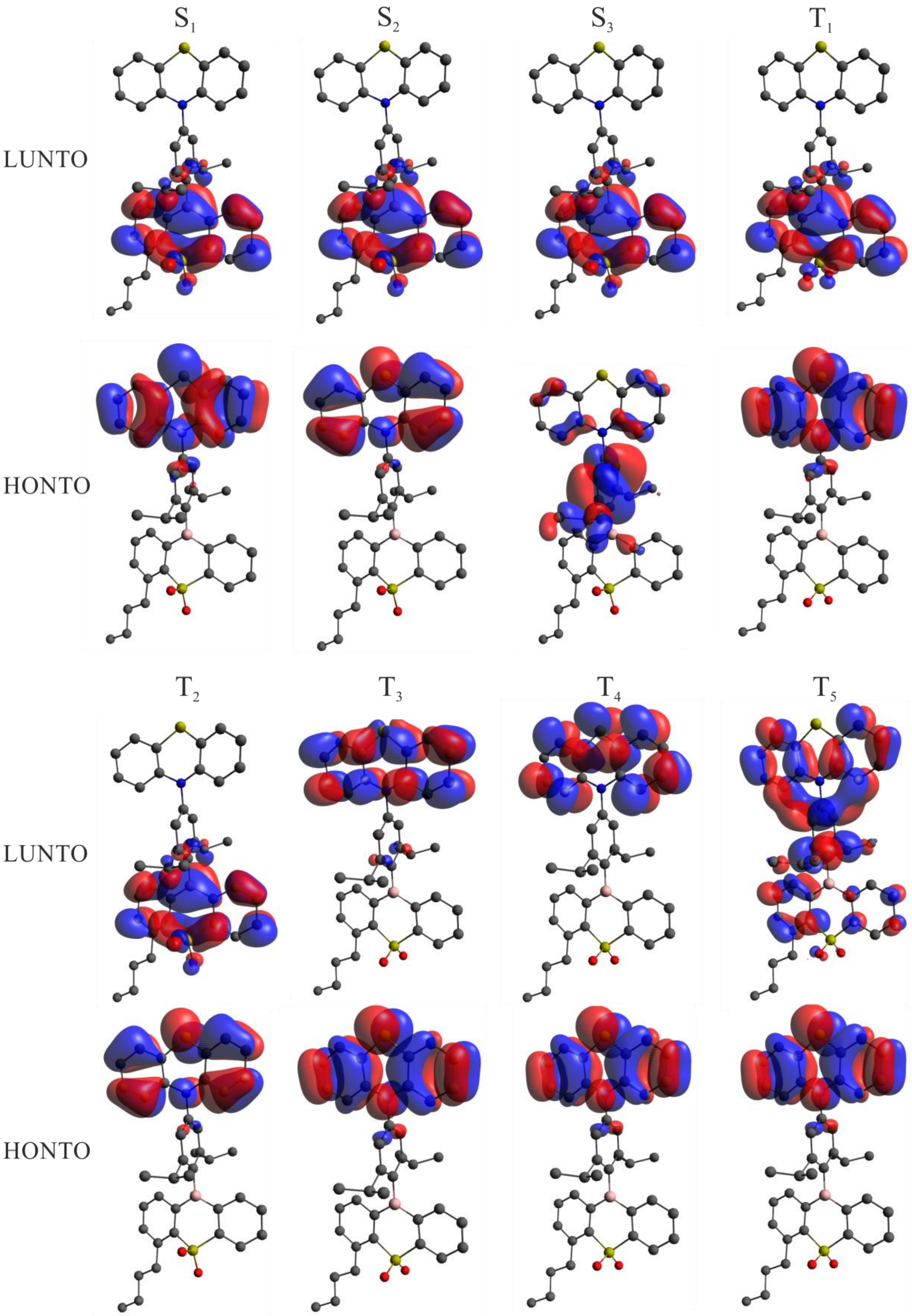

Figure S24. Natural transition orbitals for singlet and triplet states in **PTZ-Dipp-(Bu)SO2B**; *iso* = 0.02.

6. Electroluminescent devices

Table S5. Structures of OLED devices.

Device	Structure
Dev 1	ITO | HAT-CN (10 nm) | TSBPA (40 nm) | mCP(2 nm) | mCP:PO-T2T (80:20) co 7% **PTZ-Dipp-SO2B** (20 nm) | PO-T2T (50 nm) | LiF (0.8 nm) | Al (100 nm)
Dev 2	ITO | HAT-CN (10 nm) | TSBPA (40 nm) | mCP(2 nm) | mCP:PO-T2T (80:20) co 7% **PTZ-Dipp-(Bu)SO2B** (20 nm) | PO-T2T (50 nm) | LiF (0.8 nm) | Al (100 nm)
Dev 3	ITO | HAT-CN (10 nm) | TSBPA (40 nm) | **PTZ-Dipp-SO2B** (5 nm) | PO-T2T (50 nm) | LiF (0.8 nm) | Al (100 nm)
Dev 4	ITO | HAT-CN (10 nm) | TSBPA (40 nm) | **PTZ-Dipp-(Bu)SO2B** (5 nm) | PO-T2T (50 nm) | LiF (0.8 nm) | Al (100 nm)

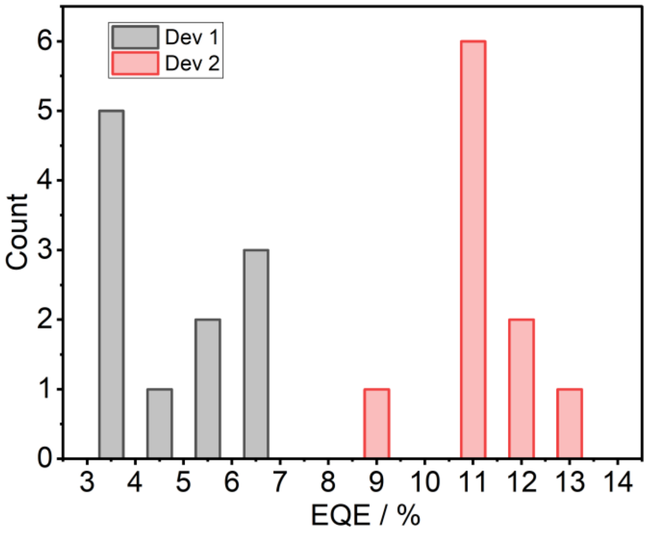

Figure S25. Distribution of maximum EQE values for the studied OLEDs with architecture of device 1 and 2.

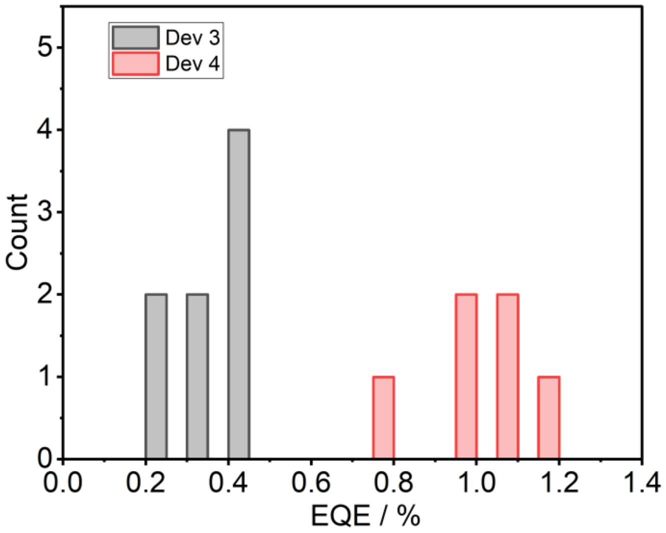

Figure S26. Distribution of maximum EQE values for the studied OLEDs with architecture of device 3 and 4.

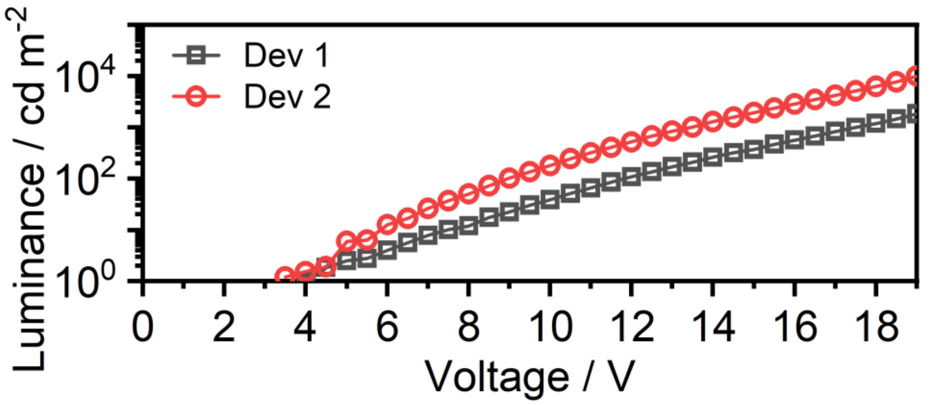

Figure S27. Luminance of devices 1 and 2 as a function of applied voltage.

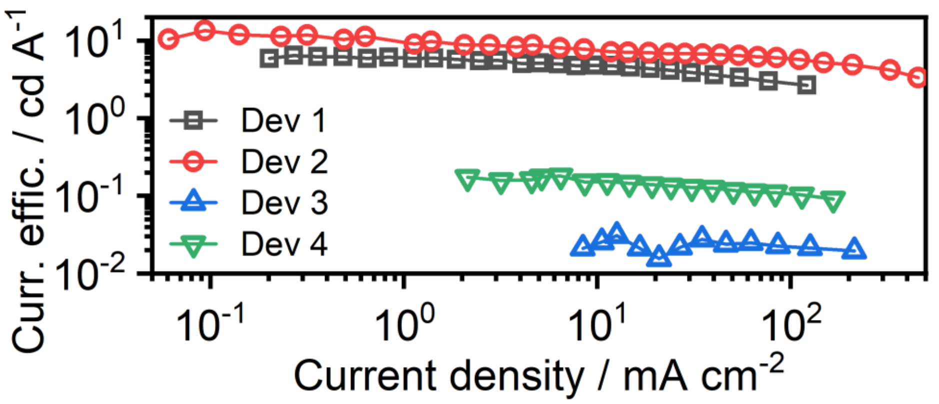

Figure S28. Current efficiency of devices 1 to 4 as a function of current density.

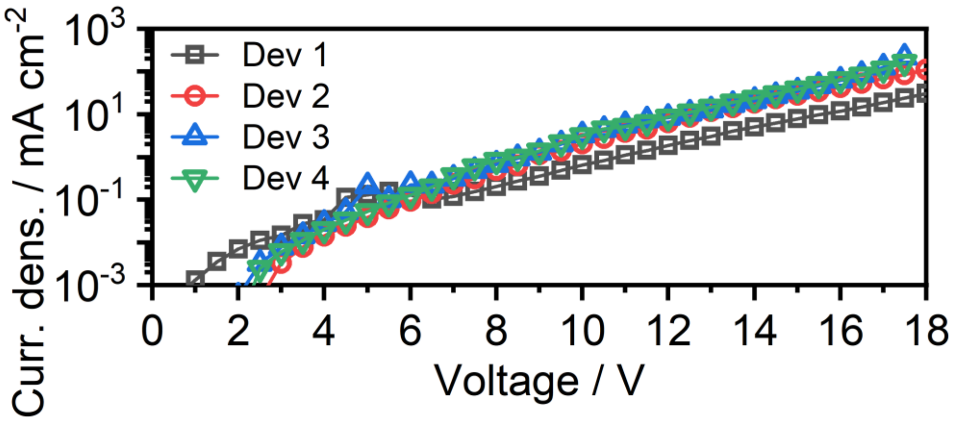

Figure S29. Current density of devices 1 to 4 as a function of applied voltage.

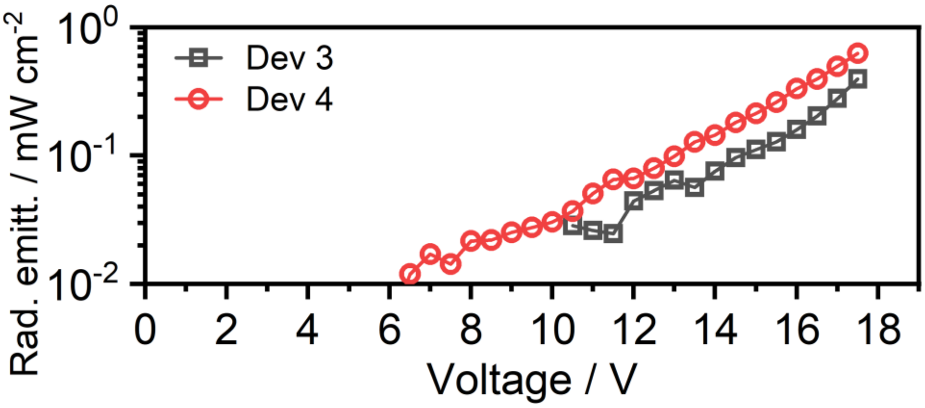

Figure S30. Radiant emittance of devices 3 and 4 as a function of applied voltage.

Table S6. Characteristics of OLED devices 1-4.

	Dev 1	Dev 2	Dev 3	Dev 4
Emitter	**PTZ-Dipp-SO2B**	**PTZ-Dipp-(Bu)SO2B**	**PTZ-Dipp-SO2B**	**PTZ-Dipp-(Bu)SO2B**
V_{ON} / V [a]	4.0	3.5	10.3	9.0
L_{max} / cd m^{-2} [b]	3300	15300	-	-
R_{max} / mW cm^{-2} [c]	-	-	0.40	0.63
λ_{EL} / nm [d]	616	601	709	681
CIE 1931 (x, y) [e]	(0.57, 0.42)	(0.54, 0.45)	(0.68, 0.33)	(0.61, 0.34)
CE_{max}/ cd A^{-1} [f]	6.5	13.6	-	-
EQE_{max} / % [g]	5.4	12.2	0.4	1.0
EQE_{max}^{av} / % [h]	5.0	11.1	0.3	0.9

[a] turn-on voltage at 1 cd m^{-2} (Devs 1 and 2) or 0.02 mW cm^{-2} (Devs 3 and 4); [b] maximum luminance; [c] maximum radiant emittance; [d] electroluminescence maxima; [e] colour coordinates of electroluminescence spectrum as defined in International Commission on Illumination colour space CIE 1931; [f] maximum current efficiency for the representative device; [g] maximum external quantum efficiency for the representative device; [h] maximum external quantum efficiency averaged over >10 independent pixels.

7. NMR and HRMS spectra

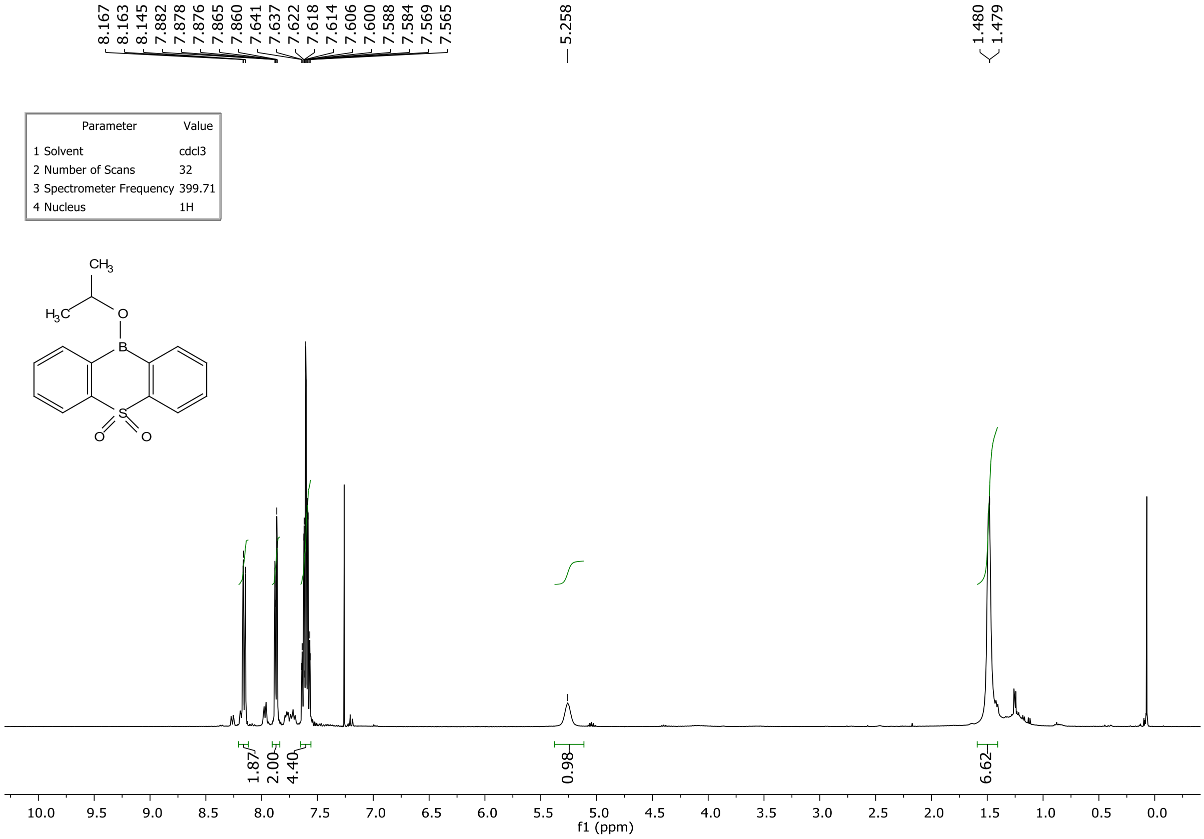

Figure S31. ^{1}H NMR spectrum of **2** (crude product) (600 MHz, $CDCl_3$).

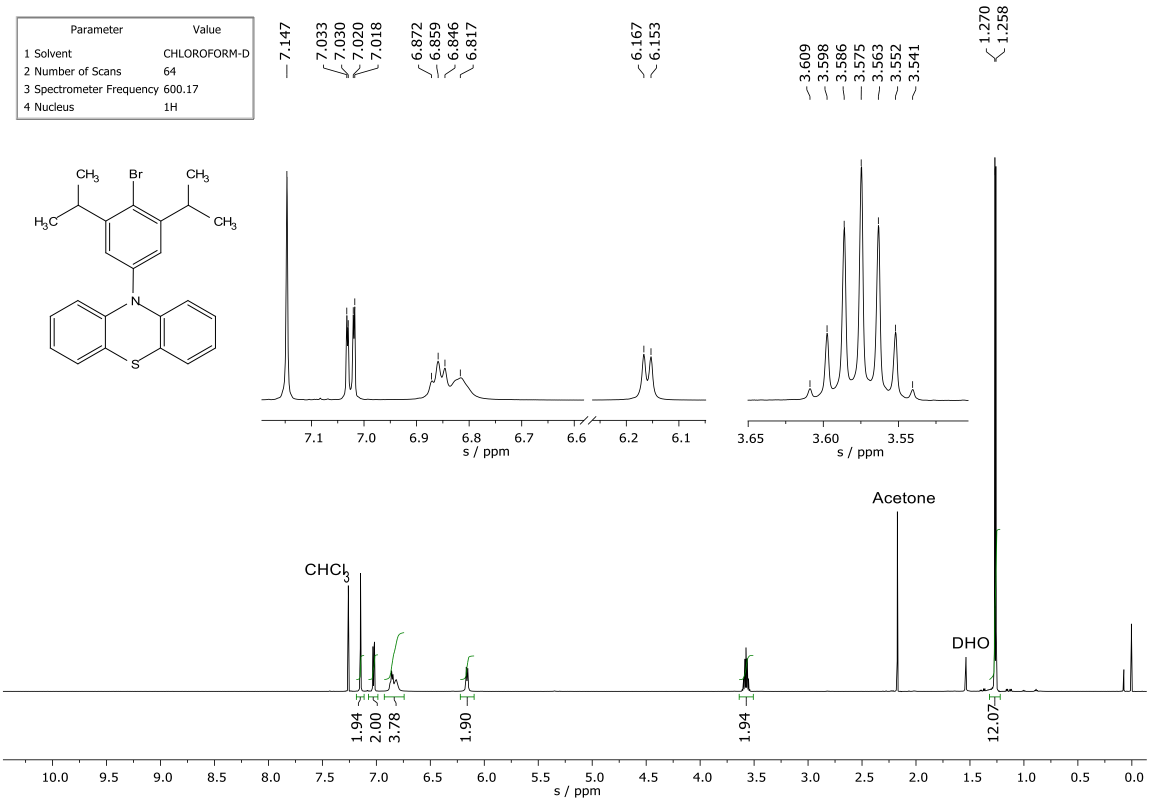

Figure S32. ^{1}H NMR spectrum of **3** (600 MHz, $CDCl_3$).

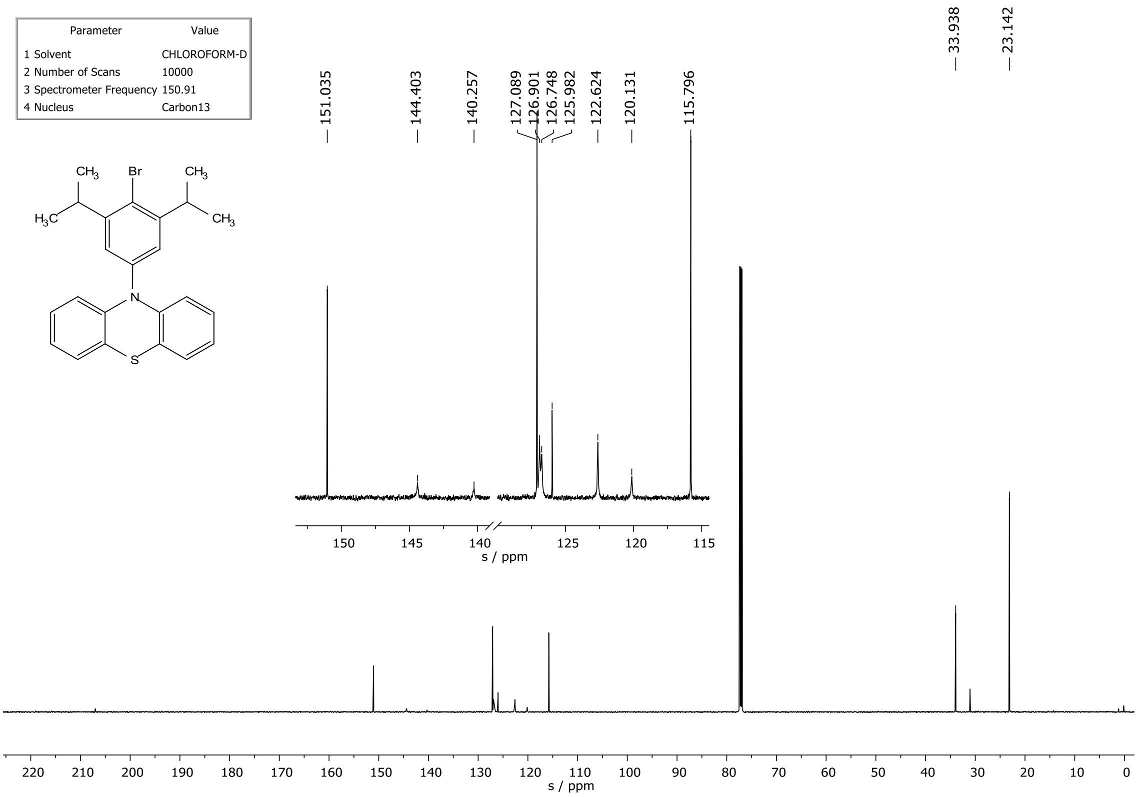

Figure S33. $^{13}C\{^1H\}$ NMR spectrum of **3** (150.9 MHz, $CDCl_3$).

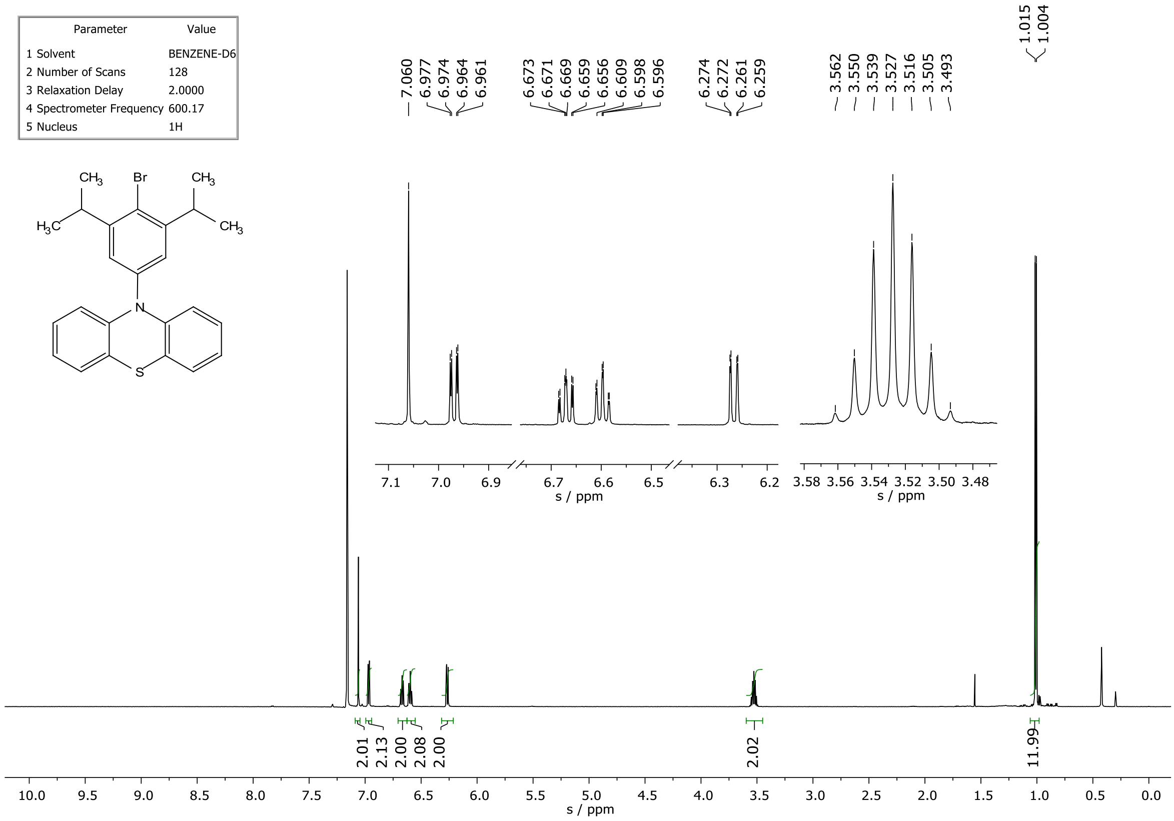

Figure S34. ^1H NMR spectrum of **3** (600 MHz, C_6D_6).

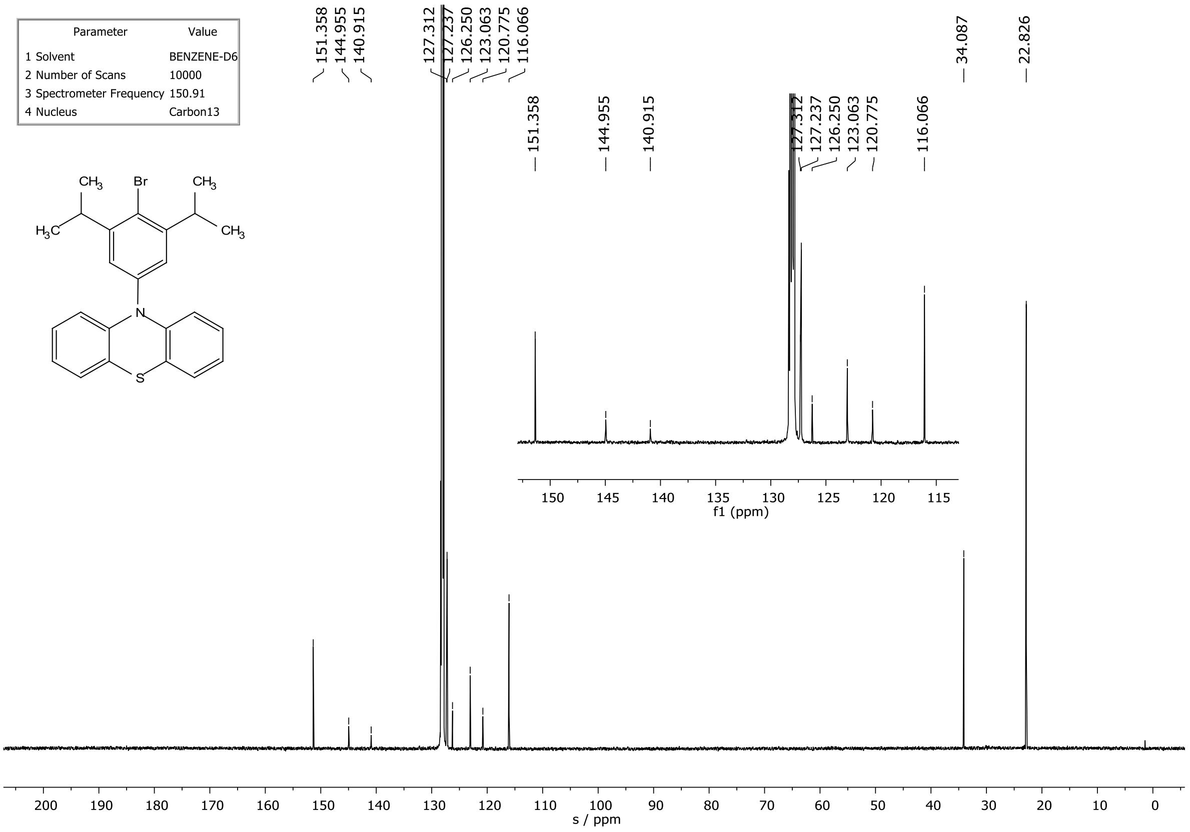

Figure S35. $^{13}C\{^{1}H\}$ NMR spectrum of **3** (150.9 MHz, C_6D_6).

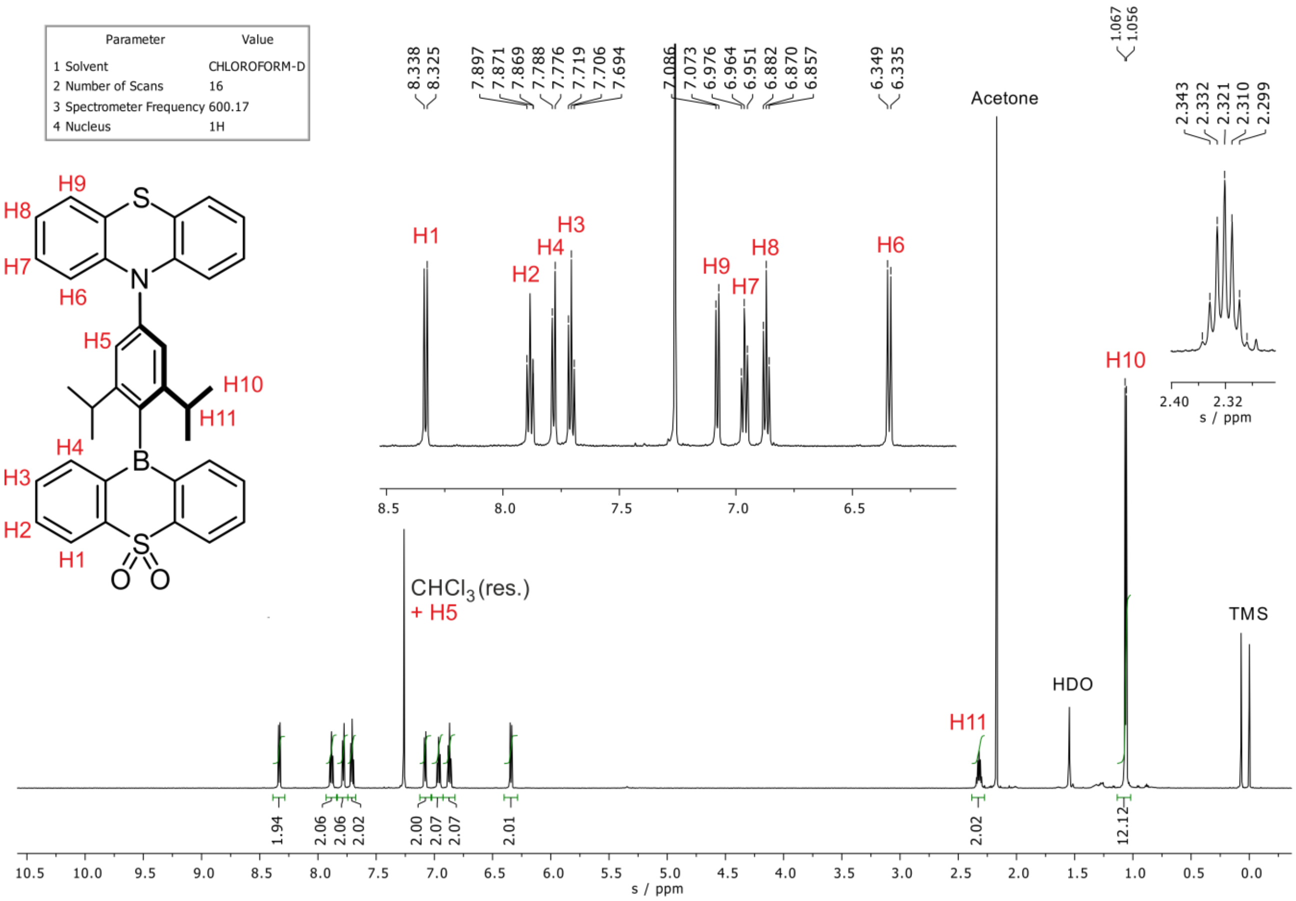

Figure S36. ^{1}H NMR spectrum of **PTZ-Dipp-SO2B** (600 MHz, $CDCl_3$).

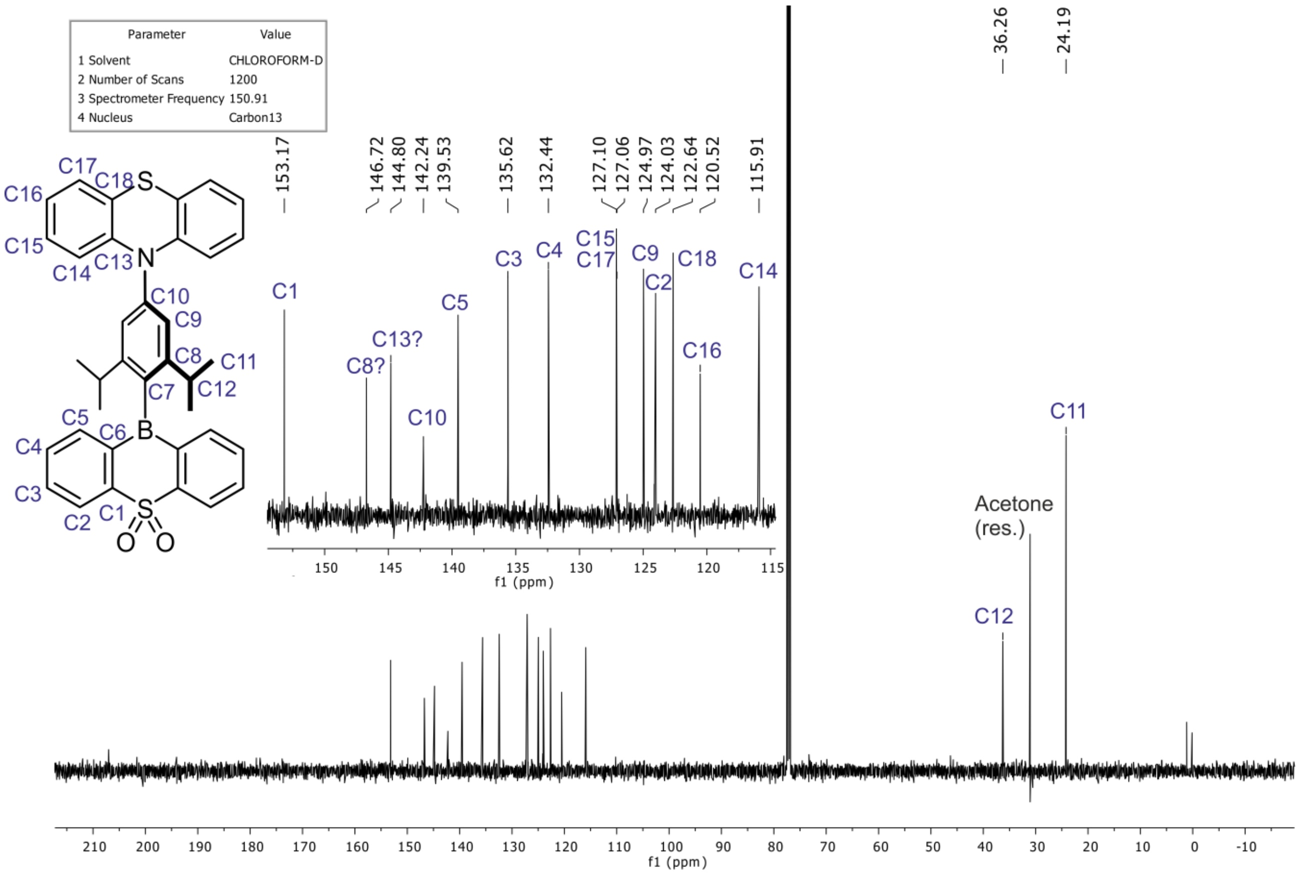

Figure S37. $^{13}C\{^{1}H\}$ NMR spectrum of **PTZ-Dipp-SO2B** (150.9 MHz, $CDCl_3$). The resonances of boron-bound carbon atoms (C6 and C7) were not observed due to their broadening by the quadrupolar boron nucleus.

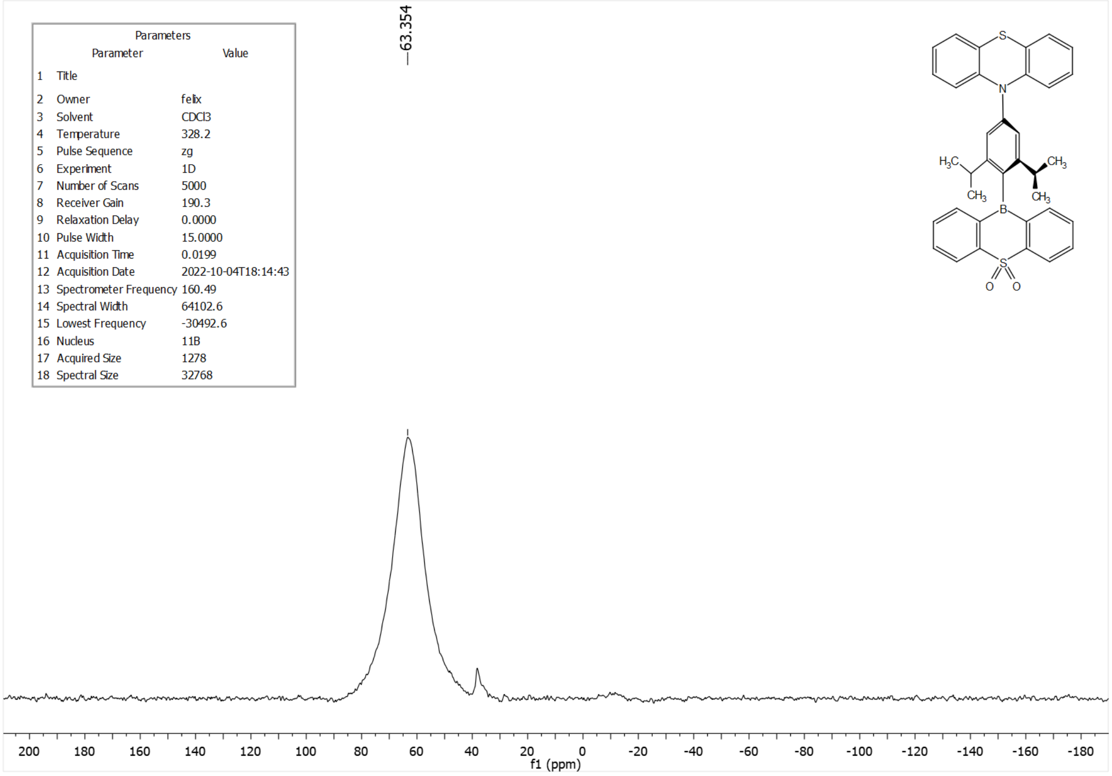

Figure S38. ^{11}B NMR spectrum of **PTZ-Dipp-SO2B** (160.5 MHz, $CDCl_3$).

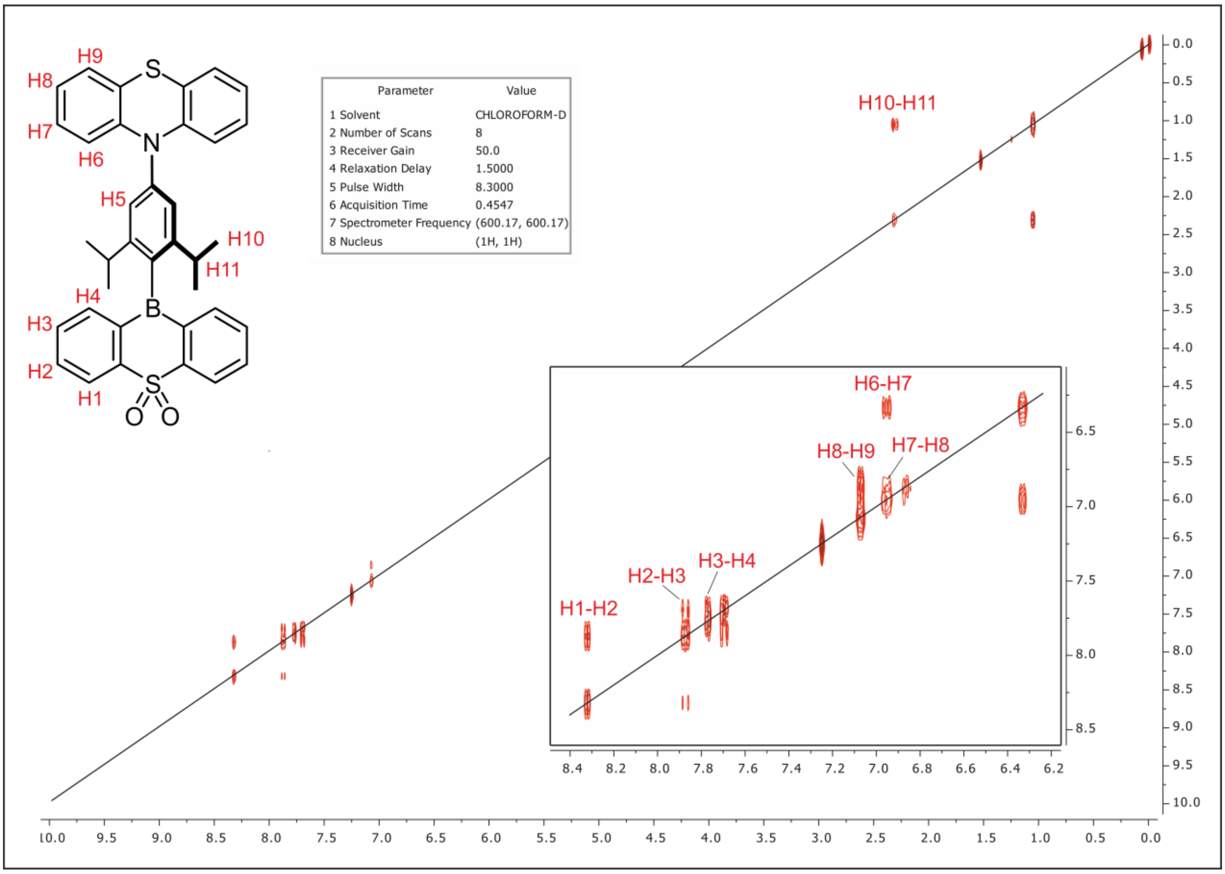

Figure S39. ^1H-^1H COSY NMR spectrum of **PTZ-Dipp-SO2B** (600 / 600 MHz, $CDCl_3$).

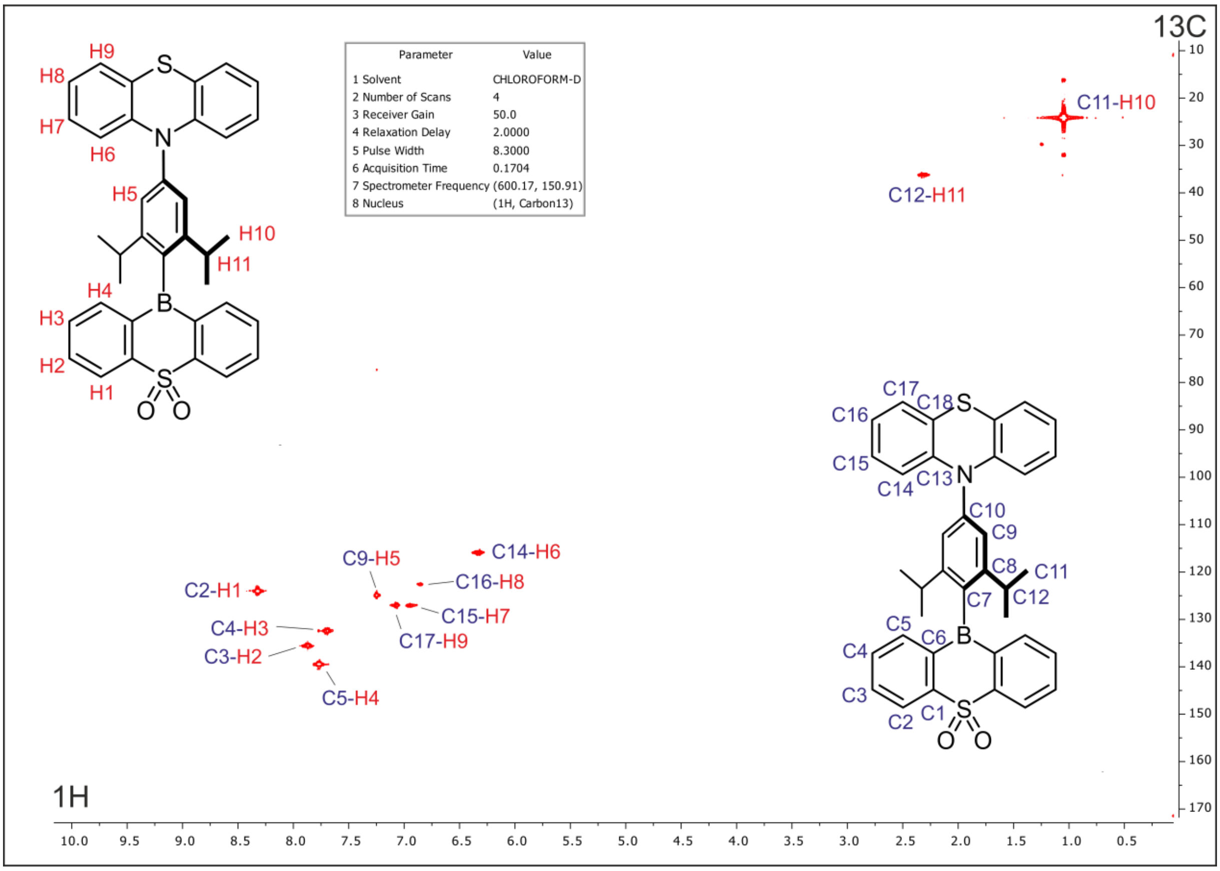

Figure S40. ^{1}H-^{13}C COSY NMR spectrum of **PTZ-Dipp-SO2B** (600 / 150.9 MHz, $CDCl_3$).

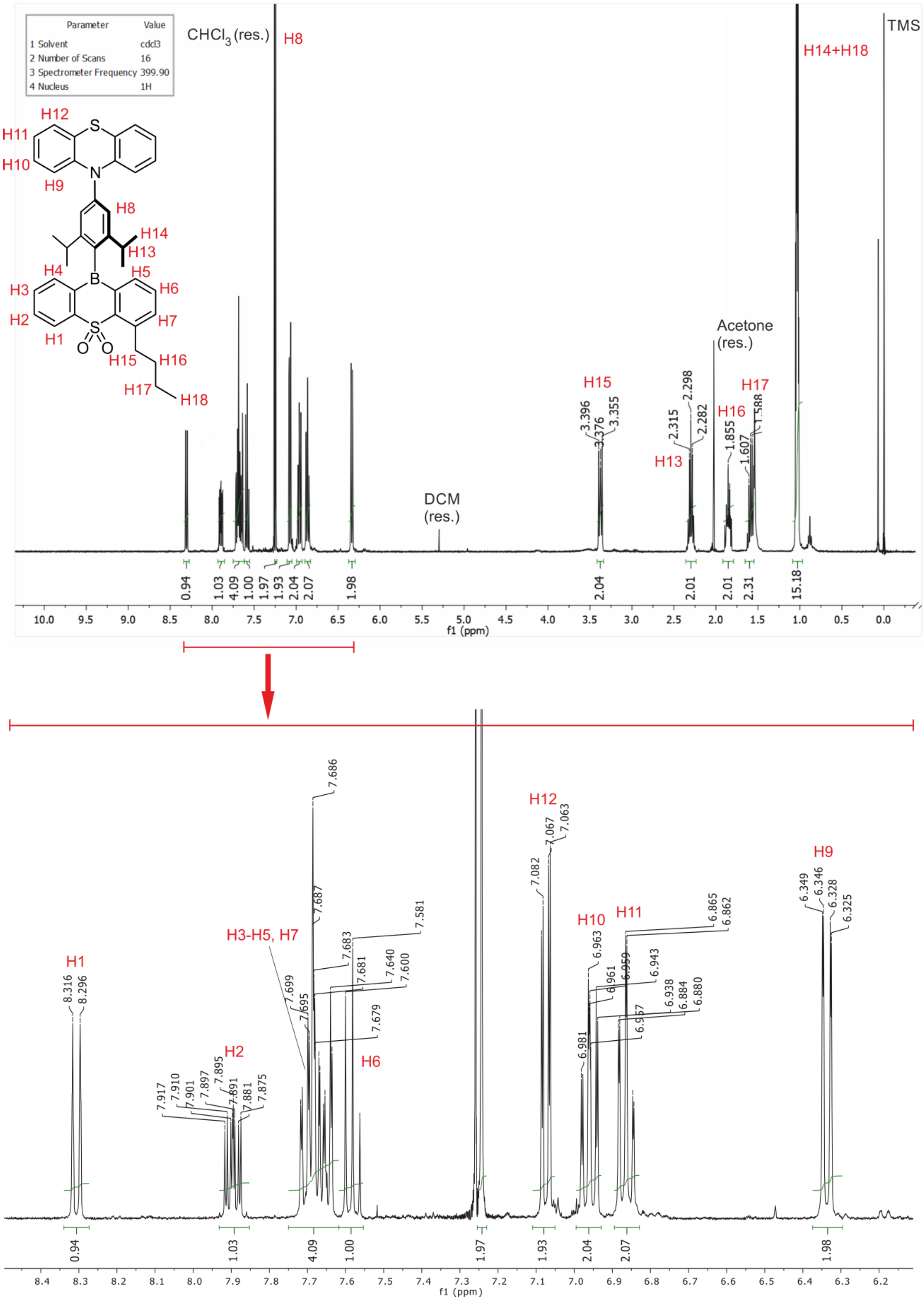

Figure S41. ^{1}H NMR spectrum of **PTZ-Dipp-(Bu)SO2B** (400 MHz, $CDCl_3$).

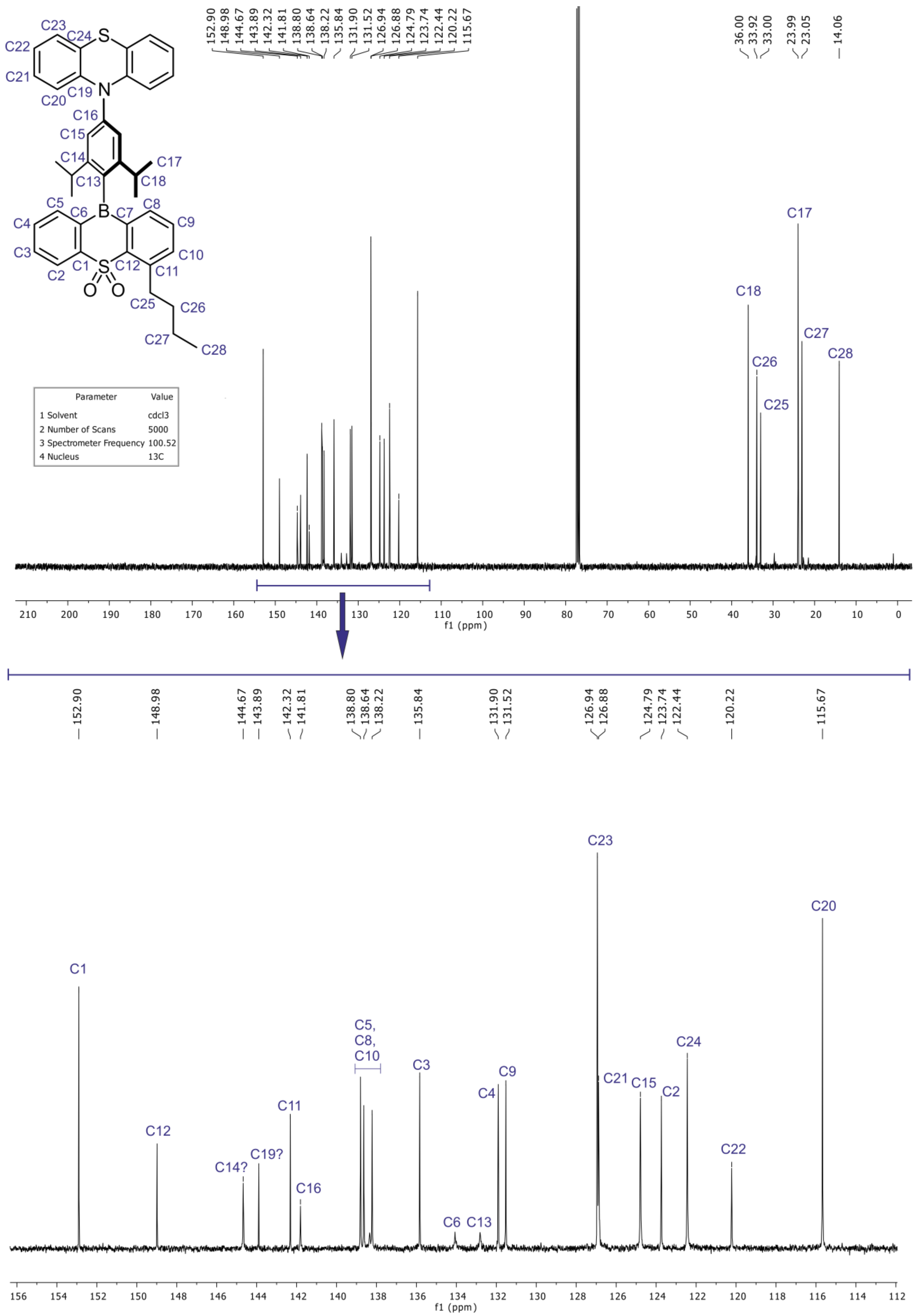

Figure S42. $^{13}C\{^{1}H\}$ NMR spectrum of **PTZ-Dipp-(Bu)SO2B** (100.5 MHz, $CDCl_3$).

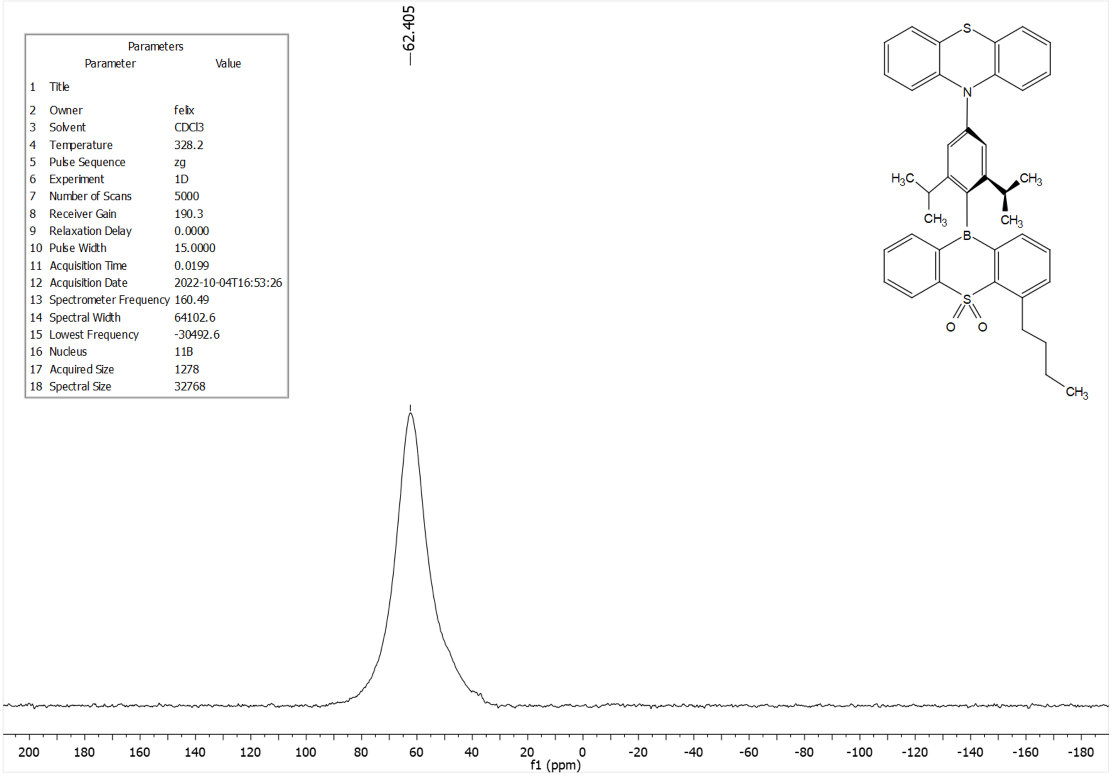

Figure S43. ^{11}B NMR spectrum of **PTZ-Dipp-(Bu)SO2B** (160.5 MHz, $CDCl_3$).

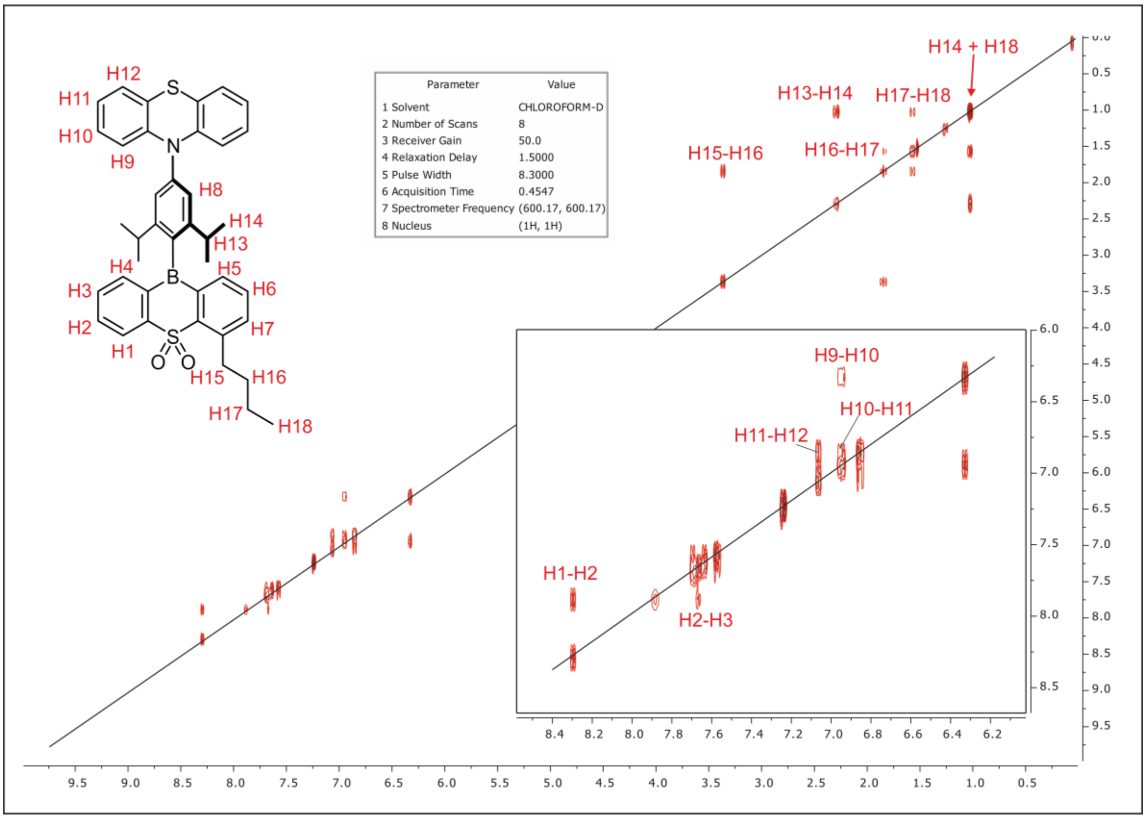

Figure S44. ^{1}H-^{13}C COSY NMR spectrum of **PTZ-Dipp-(Bu)SO2B** (600 / 150.9 MHz, $CDCl_3$).

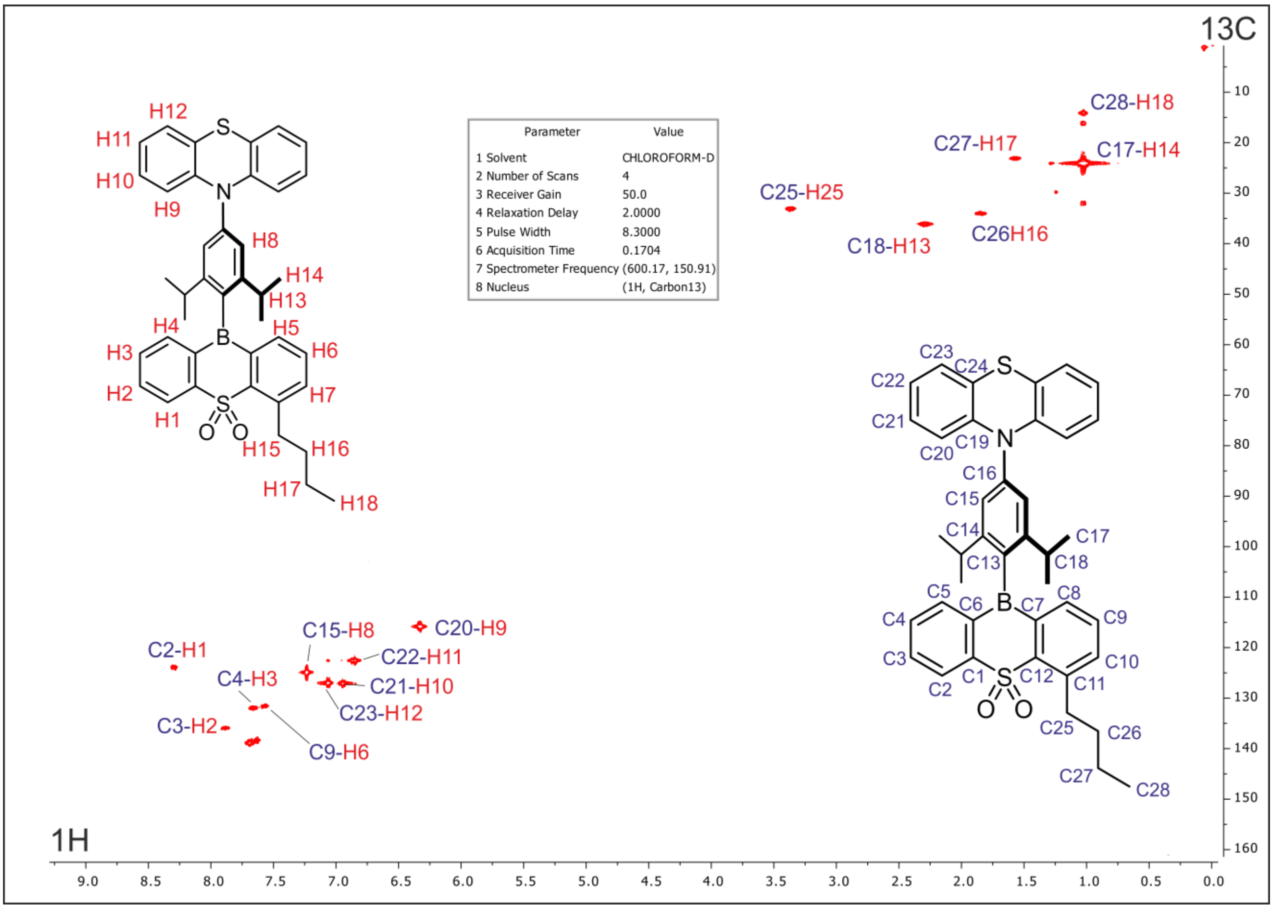

Figure S45. ^{1}H-^{13}C COSY NMR spectrum of **PTZ-Dipp-(Bu)SO2B** (600 / 150.9 MHz, $CDCl_3$).

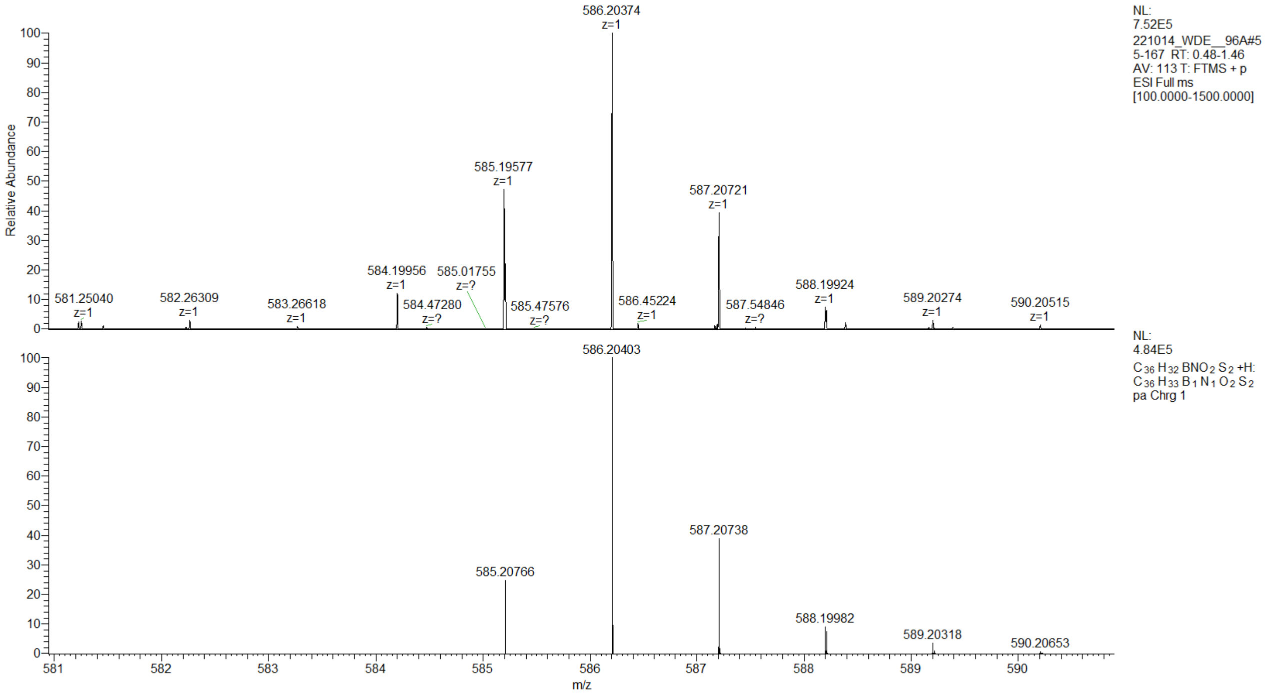

Figure S46. HRMS spectrum (ESI, positive ion mode) of **PTZ-Dipp-SO2B**. The calculated spectrum of the formula $C_{36}H_{33}BNO_2S_2^+$ $[MH]^+$ is shown at the bottom.

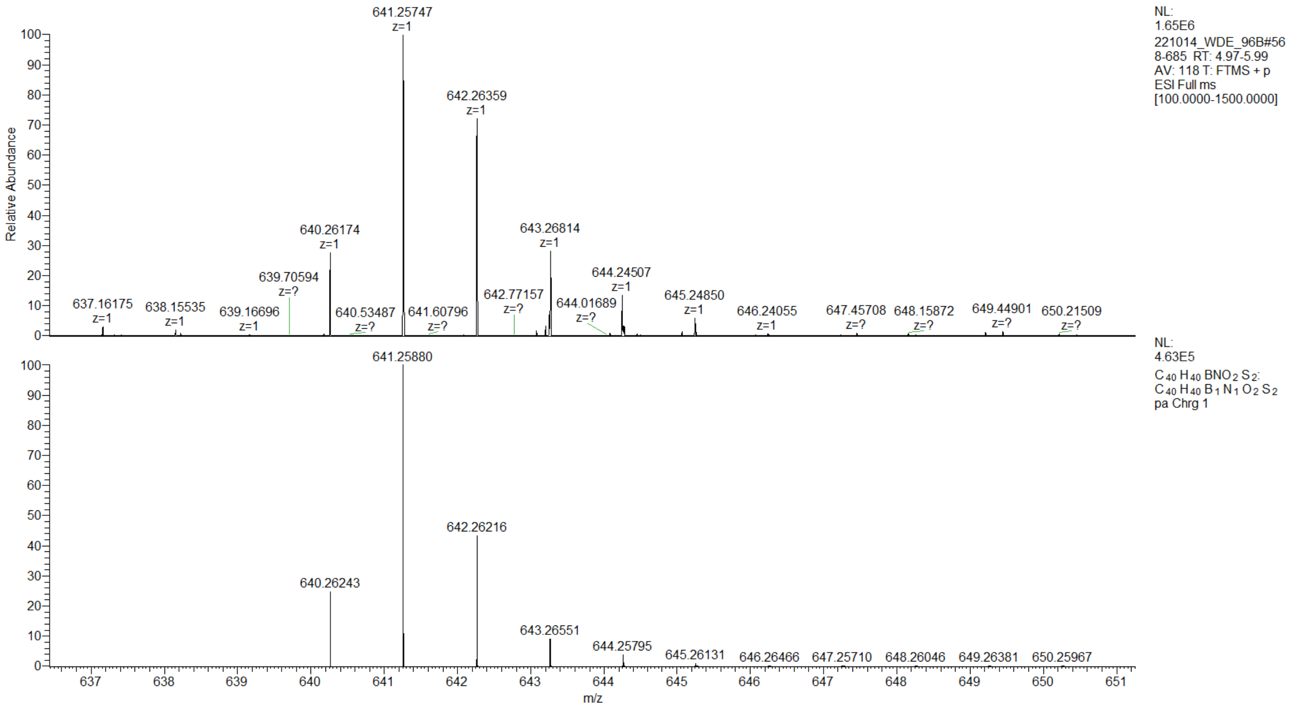

Figure S47. HRMS spectrum (ESI, positive ion mode) of **PTZ-Dipp-(Bu)SO2B**. The calculated spectrum of the formula $C_{40}H_{40}BN_2O_2S_2^+$ $[MH]^+$ is shown at the bottom.
